# Connection between Radiation-Regulating Functions of Natural Products and miRNAs Targeting Radiomodulation and Exosome Biogenesis

**DOI:** 10.3390/ijms241512449

**Published:** 2023-08-04

**Authors:** Jen-Yang Tang, Ya-Ting Chuang, Jun-Ping Shiau, Ching-Yu Yen, Fang-Rong Chang, Yi-Hong Tsai, Ammad Ahmad Farooqi, Hsueh-Wei Chang

**Affiliations:** 1 School of Post-Baccalaureate Medicine, Kaohsiung Medical University, Kaohsiung 80708, Taiwan; reyata@kmu.edu.tw; 2 Department of Radiation Oncology, Kaohsiung Medical University Hospital, Kaohsiung Medical University, Kaohsiung 80708, Taiwan; 3 Graduate Institute of Medicine, College of Medicine, Kaohsiung Medical University, Kaohsiung 80708, Taiwan; u111500006@gap.kmu.edu.tw; 4 Department of Biomedical Science and Environmental Biology, PhD Program in Life Sciences, College of Life Science, Kaohsiung Medical University, Kaohsiung 80708, Taiwan; 5 Division of Breast Oncology and Surgery, Department of Surgery, Kaohsiung Medical University Hospital, Kaohsiung Medical University, Kaohsiung 80708, Taiwan; 1060526@kmuh.org.tw; 6 School of Dentistry, Taipei Medical University, Taipei 11031, Taiwan; ycy@tmu.edu.tw; 7 Department of Oral and Maxillofacial Surgery, Chi-Mei Medical Center, Tainan 71004, Taiwan; 8 Graduate Institute of Natural Products, Kaohsiung Medical University, Kaohsiung 80708, Taiwan; aaronfrc@kmu.edu.tw (F.-R.C.); r960134@kmu.edu.tw (Y.-H.T.); 9 Institute of Biomedical and Genetic Engineering (IBGE), Islamabad 54000, Pakistan; 10 Center for Cancer Research, Kaohsiung Medical University, Kaohsiung 80708, Taiwan; 11 Department of Medical Research, Kaohsiung Medical University Hospital, Kaohsiung 80708, Taiwan

**Keywords:** natural products, miRNA, exosome, radiomodulation, targets

## Abstract

Exosomes are cell-derived membranous structures primarily involved in the delivery of the payload to the recipient cells, and they play central roles in carcinogenesis and metastasis. Radiotherapy is a common cancer treatment that occasionally generates exosomal miRNA-associated modulation to regulate the therapeutic anticancer function and side effects. Combining radiotherapy and natural products may modulate the radioprotective and radiosensitizing responses of non-cancer and cancer cells, but there is a knowledge gap regarding the connection of this combined treatment with exosomal miRNAs and their downstream targets for radiation and exosome biogenesis. This review focuses on radioprotective natural products in terms of their impacts on exosomal miRNAs to target radiation-modulating and exosome biogenesis (secretion and assembly) genes. Several natural products have individually demonstrated radioprotective and miRNA-modulating effects. However, the impact of natural-product-modulated miRNAs on radiation response and exosome biogenesis remains unclear. In this review, by searching through PubMed/Google Scholar, available reports on potential functions that show radioprotection for non-cancer tissues and radiosensitization for cancer among these natural-product-modulated miRNAs were assessed. Next, by accessing the miRNA database (miRDB), the predicted targets of the radiation- and exosome biogenesis-modulating genes from the Gene Ontology database (MGI) were retrieved bioinformatically based on these miRNAs. Moreover, the target-centric analysis showed that several natural products share the same miRNAs and targets to regulate radiation response and exosome biogenesis. As a result, the miRNA–radiomodulation (radioprotection and radiosensitization)–exosome biogenesis axis in regard to natural-product-mediated radiotherapeutic effects is well organized. This review focuses on natural products and their regulating effects on miRNAs to assess the potential impacts of radiomodulation and exosome biogenesis for both the radiosensitization of cancer cells and the radioprotection of non-cancer cells.

## 1. Introduction

Radiotherapy is a treatment for cancer in addition to chemotherapy and surgery. Radiation is an effective way to cure cancer development; however, it may incur damage to non-cancer tissues and cells, causing side effects. The efficiency of radiomodulators, such as radioprotectors and radiosensitizers, is constantly being improved to prevent tumor growth and migration and avoid side effects on non-cancer tissues [1]. Although radiation is a powerful therapy for the inhibition of cancer malignancies, improving the overall benefits of cancer therapy by protecting non-cancer cells from radiative effects with radioprotectors is desirable.

Several natural and chemical compounds have been developed as radioprotectors. Since some natural products may exhibit fewer toxic effects than compounds from chemical synthesis, they have become preferable candidates as radioprotectors [2,3]. Recently, several studies have targeted natural bioactive substances as radioprotectors [2,4,5,6,7,8,9,10,11,12,13,14,15,16,17]. However, there is a lack of understanding regarding the mechanisms of action for these radioprotective natural products.

**Figure 1:**
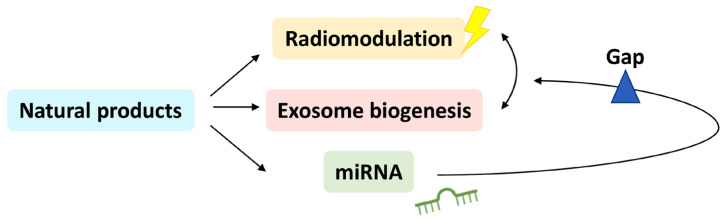
The potential relationship between natural products, their associated miRNAs, radiomodulation, and exosome biogenesis. Natural products may regulate radiomodulation, exosome biogenesis, and miRNA responses. Moreover, there may be an interplay between radiomodulation and exosome biogenesis. However, there is a knowledge gap regarding the connection between miRNAs and radiomodulation/exosome biogenesis in natural product treatments. Radiomodulation attributes radioprotection to non-cancer tissues and has radiosensitizing effects on cancer tissues. This review focuses on radioprotective natural products and their potential miRNA changes to assess the potential impacts of radiomodulation and exosome biogenesis, because some radioprotective natural products may also possess radiosensitizing effects.

Radiation response is modulated by exosomes [18,19] (Figure 1). At the same time, the composition, secretion, and cell communication of exosomes are regulated by radiation [18,19] (Figure 1). Exosomes are effectively nano-sized extracellular vesicles for delivery and communication between cells. Several of their biomolecules [20,21,22] are more abundant in cancer cells than in non-cancer cells [22,23,24,25,26,27,28,29,30,31], indicating that exosomes assist in regulating cancer growth [20,32], radioprotection [33,34], radiosensitivity [35,36], drug resistance [37], invasion [38], and metastasis [39]. Exosome biogenesis is proportional to the degree of regulation for exosomal assembly and secretion [40,41,42,43,44,45,46,47,48].

In addition to proteins and lipids, exosomes are also rich in nucleic acids [49]. Among non-coding RNAs, the present review focuses on micro-RNAs (miRNAs) within exosomes, namely exosomal miRNAs, which are short oligonucleotides (~21–23 nt) that generally target the 3′ untranslated region (3′ UTR) of responsive genes that regulate their target gene expressions. Several reports have mentioned that natural products target specific miRNAs [50,51] that regulate diverse cellular responses. However, the potential role of natural-product-regulated miRNAs in the radiation- and exosome biogenesis-modulating effects of natural products has not been investigated in detail (Figure 1).

Natural products may regulate several specific miRNAs to target many downstream signaling genes and thus modulate diverse cell functions by the natural-product–miRNA–downstream axis. This review focuses on downstream responses for radiation and exosome biogenesis in natural product treatments (Figure 1). Some literature reports have shown that several natural products are radiomodulators [2,4,5,6,7,8,9,10,11,12,13,14,15,16,17], and others offer miRNA modulation for radiation [7,52,53,54,55,56,57,58,59,60,61,62,63,64,65,66,67,68,69,70,71,72,73,74,75,76,77,78,79,80,81,82,83,84,85,86,87,88,89,90,91,92,93,94,95,96,97,98,99,100,101,102,103] and exosome biogenesis [93,104,105,106,107,108,109,110,111,112,113,114,115,116,117,118,119,120,121,122,123,124,125,126,127,128,129,130,131,132,133,134,135,136,137,138,139,140,141,142,143,144,145,146,147,148,149,150,151,152,153,154,155,156,157,158,159,160,161,162,163,164,165,166]. However, the natural-product–miRNA–downstream axis has two main knowledge gaps regarding the connections between natural products and miRNA regulation and between miRNA and the modulation of radiation and exosome biogenesis (Figure 1).

The first gap relates to the fact that radiomodulation and miRNA changes have only been reported by separate studies. The relationship between radiomodulation and miRNAs in natural product treatments has not been investigated. The second gap concerns the fact that several miRNAs have radiation-modulating effects, but examining their downstream targets with a focus on radiation- and exosome biogenesis-modulating targets has been rare. Notably, many targets related to radiation and exosome biogenesis modulation have not been investigated in these natural product and miRNA studies.

In this review, we introduce bioinformatic tools (miRDB [167] and Gene Ontology in the Mouse Genome Database (MGD) [168]) to address these knowledge gaps. miRDB [167] can provide miRNA target prediction using a bioinformatics tool, MirTarget, based on thousands of miRNA–target interaction reports and machine learning for common miRNA binding features. MGD [168] is the authoritative database for biological reference information related to gene functions and phenotypes for several diseases. The Gene Ontology function in MGD [168] provides comprehensive gene signaling information for specific cell functions and responses, such as radiation and exosome biogenesis. miRDB [167] provides a straightforward search for targets by inputting miRNA names. The combination of Gene Ontology and miRDB allowed the natural-product-regulated miRNAs to easily retrieve their potential radiation and exosome biogenesis targets and fill these gaps. The bioinformatic application is described in detail later.

This review connects radioprotective natural products to miRNAs (Section 2). Their radiomodulating potential for non-cancer and cancer cells was assessed by a detailed literature search. Next, the potential roles of radiation- (Section 3) and exosome biogenesis- (Section 4) modulating effects in these miRNAs acting on non-cancer and cancer cells were explored by performing PubMed/Google Scholar, Gene Ontology [168], and miRDB [167] bioinformatic searches. Finally, the target genes and an overview of radiation- (Section 5) and exosome biogenesis- (Section 6) modulating effects are presented. In short, this review explores the connection between and miRNA basis of the radiation- and exosome biogenesis-modulating effects of radioprotective natural products, which may also have radiosensitizing effects on cancer cells. The interconnection of the radiation-induced regulation of exosomes and the cellular processes that govern extracellular vesicle biology is essential to shed light on the functionalities of these vesicles. There is an extraordinary wealth of information about the radiation-mediated release of non-coding-RNA-loaded exosomes from donor cells.

## 2. Connection between Radioprotective Natural Products and miRNAs

Several natural products with radioprotective abilities have been studied and reported (Table 1) [2,4,5,6,7,8,9,10,11,12,13,14,15,16,17]. The radioprotective mechanism of these natural products has not yet been described in detail, particularly for miRNA modulation. Here, we focus on filling the knowledge gap regarding the connection between radiation modulation and miRNAs from natural products.

Recently, several natural product studies highlighted the importance of miRNAs in terms of their biological effects, but they did not focus on their radiation-modulating functions. Although radioprotective natural products (Table 1) have been mentioned in several literature reports, the participation of miRNAs in regulating the radiation response of cancer cells lacks a detailed investigation. The potential of the miRNA-modulating effects of these radioprotective natural products needs further assessment through a literature search and subsequent experimental investigations.

According to our PubMed/Google Scholar search, some miRNA changes have been reported in several radioprotective natural product treatments, but these studies did not investigate the radiation-modulating effects of these miRNAs (Table 1).

According to the literature search, some studies on natural products related to non-cancer radiation are available (Section 2.1), while others have investigated natural products in relation to cancer radiation (Section 2.2). Some natural products have been included in both non-cancer and cancer radiation studies (Section 2.3). Notably, some natural products addressed in non-cancer radiation studies have been reported in cancer radiation studies and vice versa, but this review cannot consider all of them. They are listed in Table 1.

### 2.1. Function of Radioprotective Natural Products in Non-Cancer Tissue Studies

Several natural products regulate the expression of certain miRNAs in non-cancer tissues, but their radiomodulating effects have not been examined in the literature, as shown below. In non-cancer radiation studies, several radioprotective natural products (Table 1) [2,5,6,9,12], such as apigenin, berberine, celastrol, chlorogenic acid, daidzein, diosmin, melatonin, silymarin, thymol, troxerutin, vitamin C, and zingerone, have been shown to avoid side effects on non-cancer tissues. These radioprotective natural products and their miRNA changes have been reported individually. Moreover, the connection between radioprotective natural products and miRNA function has not been investigated in detail.

Below, we summarize these natural products and their miRNA responses in several non-cancer tissues. Apigenin (10 μg/mL), a plant-derived trihydroxyflavone, protected lymphocytes from 3 Gy irradiation-induced DNA damage [169]. Apigenin upregulated miR-15a-5p to attenuate methotrexate-triggered neuroinflammation in rat models [170] (Table 1). Baicalein (20 mg/kg), a Chinese herb (*Scutellaria baicalensis*)-derived flavonoid, alleviated the radiation (2 Gy)-induced lung injury of lung cancer patients [10]. Baicalein inhibited liver cancer cell proliferation by upregulating miR-3178, reversed by downregulating miR-3178 [172]. Celastrol (1.5 μM), a *Tripterygium wilfordii*-derived triterpenoid, reversed 20 Gy irradiation-induced kerotinocyte antiproliferation [12]. Celastrol alleviated oxygen–glucose deprivation and the reoxygenation-induced apoptosis of brain microvascular endothelial cells by downregulating miR-6085, reverted by miR-6085 mimics [174] (Table 1). Chlorogenic acid (4 μg/mL), a fruit- and vegetable-derived phenolic derivative, protected lymphocytes from 2 Gy irradiation-induced genetic damage [179]. Chlorogenic acid suppressed H_2_O_2_-triggered oxidative stress and endoplasmic reticulum stress in hepatocytes by downregulating miR-199a-5p [180]. Daidzein (8 μM), a soybean-derived phytoestrogen isoflavone, showed photoprotective effects on skin fibroblasts irradiated by 60 mJ/cm^2^ UVB [5]. Dietary daidzein blocked hepatitis C virus replication by suppressing miR-122-5p [183].

Diosmin (200 mg/kg), a flavonoid derived from the Japanese pagoda tree, reduced DNA damage in rats irradiated with 10 Gy [184]. Diosmin can reduce some side effects. For example, gentamicin caused nephrotoxicity by downregulating miR-21-5p expression and upregulating miR-155-5p expression, which was reversed by diosmin and showed opposite results to miR-21-5p and miR-155-5p regulation [181] (Table 1). Additionally, diosmin alleviated radiation-induced hepatic fibrosis by downregulating miR-17-5p [188]. Melatonin alleviated DNA damage in the spleen and cerebral cortex of mice irradiated with 5 Gy [191]. Melatonin suppressed the endoplasmic reticulum (ER) stress-promoting hepatic steatosis of primary hepatocytes by downregulating miR-23a-3p [193] (Table 1). Silymarin (70 mg/kg for 3 days) enhanced the survival of mice irradiated with 9 Gy [5]. Silymarin attenuated thioacetamide-induced liver damage by upregulating miR-122-5p, miR-192-5p, and miR-194-5p [194] (Table 1).

Thymol (80 mg/kg), a monoterpene derivative, alleviated ovarian damage in rats irradiated with 3.2 Gy [198]. Thymol alleviated bleomycin-induced pulmonary fibrosis in mice by upregulating miR-29a-5p [199] (Table 1). Troxerutin (175 mg/kg), a flavonoid, inhibited lipid peroxidation in liver and spleen tissues of tumor-bearing mice irradiated with 4 Gy [201]. Troxerutin attenuated myocardial ischemia and reperfusion injury-triggered apoptosis by downregulating miR-146a-5p [202]. Troxerutin exhibited radioprotection for normal epithelial cells by downregulating miR-147a, which is upregulated by radiation [203]. Vitamin C (3 g/kg) increased the survival of mice irradiated with 8 Gy [204]. Vitamin C upregulated miR-215-3p, miR-215-5p, miR-371b-5p, and miR-181a-5p and downregulated miR-29b-1 and miR-589-5p in human bone marrow stromal cells [205] (Table 1). Vitamin C downregulated miR-451a in type 2 diabetes mellitus patients [210]. Zingerone (10 μg/mL) reduced the 2 Gy irradiation-induced DNA damage of lymphocytes [214]. Zingerone enhanced osteoblast differentiation by upregulating miR-200c-3p in human bone mesenchymal stem cells [215] (Table 1).

Consequently, several radioprotective miRNA candidates have been retrieved from radioprotective natural products (Table 1). Modulating these miRNA candidates revealed their radioprotective functions to avoid potential side effects on non-cancer tissues and cells.

### 2.2. Functions of Radioprotective Natural Products in Anticancer Studies

Several natural products regulate the expression of certain miRNAs in cancers, but their radiomodulating effects have not been examined as of yet. Some natural products exhibit both radioprotective and anticancer effects on non-cancer tissues and cancers, respectively. In cancer radiation studies, several radioprotective natural products with anticancer effects have been presented (Table 1) [2,4,5,6,9,10,11,14,15,16], such as baicalein, betulinic acid, caffeic acid phenethyl ester (CAPE), carvacrol, curcumin, 3,3′-diindolylmethane, emodin, fucoidan, gallic acid, genistein, hesperidin, mangiferin, matrine, melatonin, parthenolide, quercetin, rutin, sesamol, ursolic acid, and withaferin A. Different studies have individually reported these radioprotective natural products and their miRNA changes. Moreover, the connection between radioprotective natural products and miRNA function has rarely been mentioned in earlier reports.

Below, we collate these natural products and their miRNA responses in several cancers (Table 1). Berberine (300 mg × 3 time/day), a plant alkaloid, alleviated 36 Gy (1.8 Gy/fraction) irradiation-induced acute radiation intestinal syndrome in lymphoma patients [9]. Berberine upregulated miR-182-5p in mouse astrocytes to suppress neuroinflammation [218]. Betulinic acid (8 μg/mL), a plant-derived pentacyclic triterpenoid, enhanced the 4 Gy irradiation-induced antiproliferation of head and neck cancer cells [221]. Betulinic acid caused the antiproliferation and apoptosis of colon cancer cells by downregulating miR-27a [222]. CAPE (50 μmol/kg), a propolis-extract-derived phenolic natural product, alleviated 8 Gy radiation-induced pulmonary injury in rats [230]. CAPE inhibited proliferation and drove the apoptosis of lung cancer cells by upregulating miR-3960, reverted by miR-3960 knockdown [231]. Carvacrol (80 mg/kg) attenuated ovarian damage in 3.2 Gy irradiated rats [198]. Carvacrol caused the growth inhibition and apoptosis of leukemia cells by upregulating miR-217-3p and downregulating circ-0008717 levels, which could be reversed by anti-miR-217-3p treatment [232] (Table 1). Curcumin (50 mg/kg) alleviated 50 Gy radiation-induced skin response in rats [234]. Curcumin showed anticancer effects by upregulating miR-137-3p or miR-137-5p in colon [235], miR-16-5p in breast [236], miR-98-5p in lung [237], and miR-30a-5p in prostate [238] cancer cells (Table 1). In contrast, curcumin may inhibit specific miRNAs in drug-resistant cells. For example, curcumin promoted the apoptosis of cisplatin-resistant lung cancer cells by downregulating miR-186-3p, reversed by miR-186-3p overexpression [240].

3,3′-diindolylmethane (75 mg/kg), a natural product derived from the Brassica cabbage plant, enhanced the survival of 13 Gy irradiated rats [242]. 3,3′-diindolylmethane caused the antiproliferation and G2/M arrest of breast cancer cells via miR-21-5p [182]. Emodin (30 mg/kg), a traditional rhubarb-derived anthraquinone, increased rat survival and attenuated 9 Gy radiation-induced intestinal injury [15]. Emodin suppressed the epithelial–mesenchymal transition (EMT) and metastasis of pancreatic cancer cells by increasing miR-1271-5p expression [244]. Fucoidan (100 mg/kg), a brown-algae-derived sulfated polysaccharide, showed radioprotective effects in mice against 9 Gy irradiation [245]. Fucoidan suppressed the EMT of liver cancer cells by upregulating miR-29b-3p [246] (Table 1). Fucoidan also showed antiproliferative effects on breast cancer cells by increasing miR-29c-3p and decreasing miR-17-5p [247]. Gallic acid (100 mg/kg), a plant-derived triphenolic component, alleviated the 8 Gy radiation-induced DNA damage of lymphocytes in mice [250]. Gallic acid showed antiproliferative effects on breast cancer cells by upregulating miR-182-5p and downregulating miR-21-5p [251]. Gallic acid also promoted apoptosis and suppressed the migration of human chondrosarcoma cells by increasing miR-518b expression [252].

Genistein (200 mg/kg), a legume-plant-derived isoflavone, alleviated acute myelotoxicity in 7.75 Gy irradiated mice [254]. Genistein inhibited prostate cancer cell proliferation and migration by upregulating miR-574-3p [255]. Moreover, genistein downregulated miR-27a-3p in ovarian [257], miR-155-5p in breast [258], and miR-223-3p in pancreatic cancer cells [259] (Table 1). Hesperidin (250 mg), a citrus-fruit-derived flavanone glycoside, reduced 1.5 Gy irradiation-induced genetic damage in lymphocytes of human subjects [260]. Hesperidin showed antiproliferative effects on breast cancer cells by downregulating miR-21-5p and upregulating miR-16-5p and miR-34a-5p [261]. Hesperidin also inhibited proliferation and triggered the apoptosis of lung cancer cells by upregulating miR-132-3p [262] (Table 1). Mangiferin (2 mg/kg), a *Mangifera indica*-derived glucosylxanthone, enhanced the survival of 10 Gy irradiated mice [11]. Mangiferin suppressed proliferation and triggered the apoptosis of glioma and lung cancer cells by upregulating miR-15b-5p [266] and downregulating miR-27b-3p and miR-92a-3p [267] (Table 1), respectively. Matrine (30 mg/kg), a Sophora-plant-derived alkaloid, increased the survival of 7 Gy irradiated rats [269]. Matrine promoted antiproliferative and apoptotic effects in melanoma and colon cancer cells by upregulating miR-19b-3p [270] and miR-22-3p [294], respectively. For comparison, matrine suppresses proliferation and drives the apoptosis of other cancer cells by downregulating specific miRNAs. For example, matrine showed antiproliferative effects on thyroid cancer cells by downregulating miR-21-5p [295].

Melatonin reduced the proliferation and invasion and promoted the apoptosis of glioma cells by downregulating miR-155-5p [192]. Parthenolide (5 μM) improved the viability of a 7 Gy irradiated HeLa × normal skin fibroblast hybrid cell line [272]. Parthenolide is a *Tanacetum parthenium*-derived sesquiterpene lactone. Overexpressing miR-107 enhanced the antiproliferative effects of parthenolide on lung cancer cells [273] (Table 1). In comparison, parthenolide triggered the antiproliferation and apoptosis of prostate cancer cells by downregulating miR-375-3p or miR-375-5p [275]. Quercetin (1% by weight), a plant flavonoid, alleviated 35 Gy radiation-induced skin fibrosis in mice [276]. Quercetin showed anticancer effects by upregulating let-7a-5p (breast and lung cancer) and miR-146a-5p (breast, lung, and colon cancer) and downregulating miR-21-5p (lung and prostate cancer) [277] (Table 1). Rutin (200 mg/kg), a plant pigment, alleviated 5 Gy radiation-induced brain injury in rats [279]. Rutin suppressed sorafenib-promoted drug resistance and autophagy in liver cancer cells by upregulating miR-590-5p [280]. Rutin inhibited the proliferation and triggered the apoptosis of pancreatic and breast cancer cells by upregulating miR-877-3p [281] and miR-129-1-3p [282], respectively.

Estrogen receptor alpha (ESR1)-negative breast cancer shows minor sensitivity to hormone therapy. Upregulating ESR1 can improve its therapeutic effects. Sesamol (10 μg/mL), a sesame-derived phenolic antioxidant, alleviated 2 Gy radiation-induced DNA damage in lymphocytes from human subjects [284]. Sesamol re-expressed ESR1 to inhibit ESR1-negative breast cancer cells by upregulating miR-370-3p [285]. Ursolic acid (20 μM), a pentacyclic triterpenoid, increased the cell viability of 40 Gy irradiated keratinocytes [287]. Ursolic acid enhanced the sensitivity of paclitaxel and doxorubicin to breast cancer cells by upregulating miR-149-5p [288] and miR-186-5p [289], respectively. Ursolic acid suppressed breast cancer stem cells by downregulating miR-499a-5p [292]. Ursolic acid suppressed inflammation triggered by the influenza A virus and reactive oxygen species (ROS)-induced effects in lung cancer cells by upregulating miR-34c-5p [290] (Table 1). Withaferin A (10 μM), a *Withania somnifera*-derived steroidal lactone, increased the cell viability of 4 Gy irradiated splenic lymphocytes and bone marrow cells [16]. Withaferin A upregulated let-7c-5p and let-7a-5p and downregulated miR-549a-5p, miR-1247-5p, miR-124-5p, and miR-137-5p in breast-cancer-derived mammospheres [171]. Withaferin A inhibited lung cancer cell proliferation and migration by downregulating miR-27a-3p and miR-10b-5p [173].

In brief, several anticancer miRNA candidates have been retrieved from several radioprotective natural products (Table 1). Modulating these miRNA candidates revealed the antiproliferative functions of several cancer cells. These examples suggest that some radioprotective natural products protect non-cancer tissues and have antiproliferative potential against cancer cells in terms of miRNAs. This warrants a detailed investigation into the radiosensitizing function of these radioprotective natural-product-derived anticancer miRNAs.

### 2.3. Function of Radioprotective Natural Products in Non-Cancer Tissue and Anticancer Studies

Some radioprotective natural products (Table 1) [2,4,5,6,7,13,17], such as chrysin, delphinidin, ferulic acid, ginsenoside Rg1, ligustrazine, lycopene, piperine, resveratrol, and vitamin D3, have been investigated in both non-cancer and cancer radiation studies. They may avoid side effects on non-cancer tissues and cause anticancer effects. These radioprotective natural products and their miRNA changes have been individually assessed. Moreover, the connection between these radioprotective natural products and miRNA functions has rarely been reported.

Below, we summarize these natural products and their miRNA responses in several non-cancer and cancer studies (Table 1). For example, chrysin (25 mg/kg), a propolis-derived dihydroxyflavone, alleviated the 5 Gy radiation-induced neurotoxicity of rats [175]. Chrysin inhibited the secretion of exosomal miR-92a-3p in coronary artery endothelial cells [176]. Chrysin inhibited the proliferation of gastric cancer cells by upregulating let-7a-5p, miR-9-5p, miR-22-3p, miR-34a-5p, and miR-126-3p but downregulating miR-18-5p, miR-21-5p, and miR-221-3p [177] (Table 1). Delphinidin (5 µM) enhanced the cell viability of 3 Gy irradiated normal lung cells [185]. Dietary delphinidin alleviated muscle atrophy and colon cancer metastasis by upregulating miR-23a-3p [186] and miR-204-3p [187], respectively. Epigallocatechin gallate (EGCG) (25 mg/kg) alleviated immune system damage in 6 Gy irradiated mice [189]. EGCG upregulated miR-30a-5p in cardiomyocyte-derived exosomes [190]. EGCG enhanced liver cancer cell sensitivity to radiation [7]. Ferulic acid (50 mg/kg) reduced DNA damage in 8 Gy irradiated mice [195]. Ferulic acid enhanced osteogenesis by downregulating miR-340-3p [197]. Moreover, lipopolysaccharides (LPSs) caused intestinal epithelial barrier dysfunction by downregulating miR-200c-3p. This LPS effect was reversed by ferulic acid, a plant-derived natural product that can upregulate miR-200c-3p in colon cancer cells [196] (Table 1).

Ginsenoside Rg1 (20 mg/kg), a *Panax vietnamensis*-derived natural product, attenuated 6.5 Gy radiation-promoted aging in mice [17]. Ginsenoside Rg1 alleviated irradiation-induced changes, such as oxidative stress, DNA damage, and senescence, in hematopoietic stem/progenitor cells to enhance radioprotection [17]. miR-126-3p has a low expression in lung cancer cells. Ginsenoside Rg1 inhibited proliferation and drove apoptosis in lung cancer cells by upregulating miR-126-3p [200] (Table 1). A low level of miR-126-3p enhanced blood–brain barrier (BBB) permeability and inhibited nerve apoptosis to mitigate traumatic brain injury [296]. Ginsenoside Rg1 reduced the miR-21-5p exosome content in interleukin-1β-induced macrophages, providing therapeutic potential for traumatic brain injuries [296].

Ligustrazine (40 mg/kg), a natural product derived from a Chinese herb (Chuanxiong), increased the survival of 9.5 Gy irradiated mice [206]. Ligustrazine showed inhibitory effects on ovarian cancer cell proliferation and migration by upregulating miR-211-5p, which was partly reversed by miR-211-5p knockdown [207] (Table 1). In non-cancer studies, ligustrazine also modulated the expression of several miRNAs. For example, ligustrazine enhanced the proliferation and migration and suppressed the apoptosis of hypoxia/reoxygenation-treated trophoblast cells by upregulating miR-27a-3p [208]. Ligustrazine downregulated miR-16-5p to alleviate pre-eclampsia syndrome, a severe pregnancy complication [209].

Lycopene (10 μg/mL), a carotenoid natural product, inhibited DNA damage in 4 Gy irradiated lymphocytes [211]. Lycopene showed antiproliferative and apoptotic effects on prostate cancer cells by upregulating let-7f-1-3p [212]. Lycopene inhibited atrazine-induced ROS generation and apoptosis in the B-cell zone by downregulating miR-27a-3p [213] (Table 1). Piperine (2.5 μg/mL), a black-pepper-derived alkaloid, reduced DNA damage in 1.5 Gy irradiated lymphocytes [216]. Piperine suppressed leptin-induced anti-breast-cancer effects, inhibiting proliferation and migration, and inhibited breast tumor growth in obese mice by upregulating miR-181c-3p [217]. Moreover, piperine has non-cancer modulation effects. Thioacetamide-induced liver fibrosis in mice was alleviated by piperine treatment through the downregulation of miR-17-5p [219]. Piperine alleviated myocardial ischemia/reperfusion injury by downregulating miR-383-3p and miR-383-5p [220].

Resveratrol (100 mg/kg) inhibited the chromosome aberrations of bone marrow cells in 3 Gy irradiated lymphocytes [223]. Resveratrol alleviated allergic asthma and inflammation in mouse lungs by downregulating miR-34a-5p [224] (Table 1). Resveratrol caused antiproliferative and apoptotic effects on breast cancer cells by downregulating miR-542-3p and miR-125b-5p [225]. Vitamin D3 (alfacalcidol soft capsules) increased white blood cell numbers and inhibited bone marrow micronucleus damage in 6 Gy irradiated human subjects [226]. Vitamin D3 alleviated testicular torsion by upregulating miR-145-5p [227]. Vitamin D3 showed antiproliferative effects on gastric and liver cancer cells by upregulating miR-99b-3p [228] and miR-15a-5p [297].

Consequently, some miRNA candidates modulated by several radioprotective natural products (Table 1) provide both radioprotection for non-cancer cells and antiproliferation for cancer cells. A detailed investigation into the radioprotective and radiosensitizing functions of these natural-product-modulated miRNAs is warranted.

### 2.4. Other Radioprotective Natural Products Needing Further Investigation

Searches of PubMed/Google Scholar found that miRNAs in several radioprotective natural products have rarely been reported (Table 1). This held true, for example, for esculentoside A [233], allicin [239], caffeine [241], chlorophyllin [243], dehydrozingerone [8], *N*-acetyl tryptophan glucopyranoside [248], gymnemagenin [253], psoralidin [256], quinic acid [179], vanillin [263], and zymosan A [264].

Esculentoside A (10 mg/kg), a *Phytolacca esculenta*-derived saponin, attenuated 30 Gy radiation-induced dermatitis in mice [233]. Gymnemagenin (0.3 mg/kg) increased the survival of 10.2 Gy irradiated fish (*Pangasius sutchi*) [253]. Allicin (1 μg/mL) inhibited the 8 Gy radiation-induced upregulation of intercellular adhesion molecule-1 (ICAM-1) in umbilical vein endothelial cells [239]. Caffeine (1 mM) attenuated chromosome breaks in 0.5 Gy irradiatiate lymphoblastoid cells [241]. Chlorophyllin (1 mg/mL) suppressed the DNA damage of 5 Gy irradiated lymphocytes [243]. Dehydrozingerone (100 mg/kg) alleviated radiation sickness and increased survival in 50 Gy irradiated mice [8]. *N*-Acetyl tryptophan glucopyranoside (0.25 μg/mL) increased the cell viability of 20 Gy irradiated macrophages [248]. Psoralidin (400 μM) downregulated pro-inflammatory cytokines in 6 Gy irradiated normal lung fibroblasts [256]. Quinic acid (4 μg/mL) inhibited genetic damage in 2 Gy irradiated lymphocytes [179]. Vanillin (100 μg/mL) attenuated chromosome aberrations in 12 Gy irradiated fibroblasts [263]. Zymosan A (50 mg/kg) reduced DNA damage in 8 Gy irradiated lymphocytes [264].

In addition to terrestrial biota-derived radioprotective natural products, many bioactive substances isolated from algae and invertebrates of the world’s oceans also show potential for radioprotection. Many radioprotectors have been identified from marine sources, such as macroalgae, microalgae, sponges, sea cucumber, and corals [268,271,274,278,283,286,293,298,299,300,301,302].

Dieckol, eckol, phloroglucinol, triphlorethol A, diphlorethohydroxycarmalol, and laminarans are macroalgae-derived natural products (brown algae, *Ecklonia cava*). Dieckol (10 μg/mL) showed radioprotective effects on V79-4 lung fibroblast cells against 20 Gy radiation [265]. Eckol (10 mg/kg) enhanced the survival of 9 Gy irradiated mice [268]. Phloroglucinol (20 mg/kg) increased the survival of 8 Gy irradiated mice [271]. Triphlorethol A (30 μM) prevented the antiproliferation of 20 Gy irradiated V79-4 lung fibroblast cells [274].

Moreover, radioprotectors have also been identified in other brown algae. For example, diphlorethohydroxycarmalol (100 mg/kg), isolated from the brown algae *Ishige okamurae*, enhanced the intestinal crypt survival of mice after 10 Gy irradiation [278]. Laminarans (50 μg/mL), a *Laminaria digitata*-derived compound, showed protective effects on 4 Gy irradiated normal epidermal cells [283].

Several radioprotectors have been identified from microalgae and marine animals. For example, a β-carotene supplement (40 mg), derived from the microalga *Dunaliella bardawil*, showed radioprotection by reducing oxidized conjugated dienes in the serum of children affected by the Chernobyl accident [286]. Astaxanthin (50 μg/mL), isolated from microalgae and marine animals (shrimps, sponges, and coral), enhanced the survival of 4 Gy irradiated mice [291]. Cumaside (0.01 μg/kg), a *Cucumaria japonica*-derived triterpene glycoside, increased the survival of 6.5 Gy irradiated mice [293].

Thus, a detailed assessment of the potential impact of these natural products on miRNA regulation is warranted.

## 3. Connection between Natural-Product-Regulated miRNAs and Radiation-Modulating Effects

Several studies have focused on natural products with radiation-modulating potential (Table 1). These natural products also show miRNA-modulating effects. However, the impacts of these miRNAs on the radiation-modulating function have not been investigated. This warrants a detailed assessment of the relationship between these miRNAs and radiation-modulating functions.

After an in-depth literature search, it was evident that several of the miRNAs mentioned in Table 1 showing radiation-modulating functions need further clarification. However, the literature reports have rarely assessed the participation of radiation-modulating genes. Radiation-modulating signaling is reported in the Mouse Genome Database via the Gene Oncology function (GO:0071480 and GO:0071481) [168], i.e., cellular response to gamma radiation and cellular response to X-rays (https://www.informatics.jax.org/vocab/gene_ontology/GO:0071480 and https://www.informatics.jax.org/vocab/gene_ontology/GO:0071481 (accessed on 1 June 2023)).

**Figure 2:**
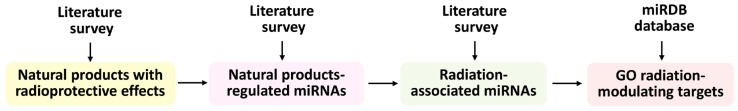
Strategy for filling the knowledge gap related to the connection between natural products, their associated miRNAs, and radiation-modulating targets. Through a PubMed/Google Scholar search, literature surveys for (1) radioprotective natural products and (2) natural-product-regulated miRNAs were performed (Table 1). Notably, several natural products were individually reported to have radioprotective and miRNA-modulating effects; however, the impact of miRNAs on the radiation response during treatment with these natural products remains unclear. (3) The radiation impact (radioprotection for non-cancer tissues and/or radiosensitivity for cancer cells) of these miRNAs was assessed by a literature search. Finally, (4) these miRNAs were fed into miRDB [167] to retrieve the GO radiation-modulating genes summarized from the Gene Ontology function in MGD (GO:0071480 and GO:0071481) [168].

As summarized in the Gene Ontology function in MGD [168], a set of genes are reported to modulate radiation response (Figure 2), including ATM serine/threonine kinase (*ATM*), ATR serine/threonine kinase (*ATR*), BCL2-like 1 (*BCL2L1*), cyclin D2 (*CCND2*), cyclin-dependent kinase inhibitor 1A (*CDKN1A*), checkpoint kinase 2 (*CHEK2*), crystallin alpha B (*CRYAB*), DNA-damage-induced apoptosis suppressor (*DDIAS*), early growth response 1 (*EGR1*), ETS transcription factor ELK1 (*ELK1*), GATA binding protein 3 (*GATA3*), general transcription factor IIH subunit 5 (*GTF2H5*), H2A.X variant histone (*H2AX*), HRas proto-oncogene, GTPase (*HRAS*), heat-shock transcription factor 1 (*HSF1*), lysine demethylase 1A (*KDM1A*), mitogen-activated protein kinase 20 (*MAP3K20*; *ZAK*), MDM2 proto-oncogene (*MDM2*), NIPBL cohesin loading factor (*NIPBL*), nuclear casein kinase and cyclin-dependent kinase substrate 1 (*NUCKS1*), proline rich acidic protein 1 (*PRAP1*), ring finger protein 4 (*RNF4*), ribosomal protein L26 (*RPL26*), secreted frizzled related protein 1 (*SFRP1*), SFRP2, tousled-like kinase 2 (*TLK2*), transmembrane protein 109 (*TMEM109*), three prime repair exonuclease 1 (*TREX1*), transformation-related protein 53 (*TRP53*), TSPY-like 5 (*TSPYL5*), WRN RecQ-like helicase (*WRN*), X-ray repair cross-complementing 5 (*XRCC5*), XRCC6, Yes1-associated transcriptional regulator (*YAP1*), and zinc metallopeptidase STE24 (*ZMPSTE24*).

To investigate the potential impact of the natural-product-regulated miRNAs (Table 1) on radiation-associated signaling (GO:0071480 and GO:0071481) [168], the miRDB [167] was applied to the target prediction of these radiation-associated miRNAs (Figure 2).

Following this strategy (Figure 2), several miRNAs associated with natural products and their potential connections to radiation-modulating effects and genes were assessed. Although these natural products are radioprotectors (Table 1; step 1), their modulated miRNAs (Table 1; step 2) were retrieved from different studies unrelated to radiation. The literature search was performed to test the potential effects of modifying these miRNAs on radiation (step 3). Notably, these miRNA candidates could impact both non-cancer and cancer cells, exhibiting radioprotection and radiosensitivity, respectively. Finally, these radiation-associated miRNAs were fed into miRDB [167] to predict the GO radiation-modulating targets (step 4).

After the literature search (Figure 2), several miRNAs were identified from non-cancer radiation studies (Section 3.1), while others were associated with cancer radiation studies (Section 3.2). Several miRNAs were associated with both non-cancer and cancer radiation studies (Section 3.3). Notably, some miRNAs investigated in non-cancer radiation studies were also reported in cancer radiation studies and vice versa, but this review cannot list them all. They are summarized in Table 2.

### 3.1. Function of Natural-Product-Regulated miRNAs in Non-Cancer Radiation Studies

For non-cancer radiation studies, several natural-product-regulated miRNAs, such as miR-17-5p, miR-518b, miR-223-3p, miR-19b-3p, miR-375-3p, miR-877-3p, miR-147a, miR-34c-5p, and miR-124-5p, were identified (Table 2) [52,53,54,55,56,57,58,59]. These miRNAs may be biomarkers for monitoring radiation toxicity in non-cancer tissues or function as radiomodulators. Modulating these miRNAs may have the potential to provide radioprotection for non-cancer tissues. The impacts of natural-product-regulated miRNAs on the radiation responses of non-cancer cells are highlighted below (Section 3.1.1 and Section 3.1.2).

#### 3.1.1. Some Natural-Product-Regulated miRNAs Are Highly Expressed in Non-Cancer Radiation Studies

In general, each miRNA may have hundreds of predicted target genes according to miRDB. The potential radiation-modulating genes were retrieved from the target search results after inputting natural-product-modulating miRNAs. Consequently, the potential radiation-modulating gene targets for the natural-product-modulating miRNAs were individually identified. The bioinformatic target prediction of radiation-modulating genes for each natural-product-modulating miRNA was performed using miRDB [167]. Some natural-product-regulated miRNAs in non-cancer tissues are upregulated by radiation (Table 2) [52,53,54,55,56,57], and their potential radiation-modulating targets are described below. All the potential radiation-modulating targets according to miRDB are referred to as radiation targets.

For example, radiation upregulated miR-17-5p in peripheral mononuclear blood cells [52], and its radiation targets *DDIAS*, *MAP3K20*, and *CCND2* were identified. miR-518b was upregulated in endothelium-derived cells after irradiation [53], and its radiation target *EGR1* was identified (Table 2). Radiation downregulated miR-223-3p in the mouse liver [95] and human peripheral mononuclear blood cells [52], and its radiation target *MDM2* was identified (Table 2). Radiation upregulated serum miR-375-3p in mice [55], and its radiation target *MAP3K20* was identified (Table 2). Light-emitting diode (LED) irradiation enhanced the proliferation of cardiomyocytes by upregulating miR-877-3p, which was reversed by miR-877-3p knockdown [56]. The radiation targets of miR-877-3p, *GTF2H5*, *KDM1A*, and *WRN,* were identified (Table 2). Radiation upregulated miR-147a in lung fibroblasts [57], and its radiation target *MDM2* was identified (Table 2). UVB upregulated miR-34c-5p to promote the UVB-induced premature senescence of dermal fibroblasts, which was reversed by miR-34c-5p knockdown [54]. The radiation targets of miR-34c-5p, *TMEM109* and *GATA3*, were identified (Table 2).

Consequently, these reports suggest that these natural products may have radiation-modulating effects on non-cancer cells in relation to their radiation-modulating genes predicted based on miRNA.

#### 3.1.2. Some Natural-Product-Regulated miRNAs Can Function as Radioprotectors in Non-Cancer Tissues

Some natural-product-regulated miRNAs in non-cancer tissues may function as radioprotectors (Table 2) [58,59]. For example, rosmarinic acid alleviated radiation-induced pulmonary fibrosis by suppressing inflammation and ROS levels via the upregulation of miR-19b-3p [58], and its radiation targets *CCND2* and *MAP3K20* were identified (Table 2). Exosomal miR-124-5p overexpression alleviated the radiation-induced cognitive dysfunction and microglial activation of the irradiated brain [59], and its radiation targets *DDIAS*, *YAP1*, and *TLK2* were identified (Table 2).

Consequently, these reports suggest that these natural products may have radioprotective effects on non-cancer cells in relation to their radiation-modulating genes predicted based on miRNA.

### 3.2. Function of Natural-Product-Regulated miRNAs in Cancer Radiation Studies

Several natural-product-regulated miRNAs, such as miR-15a-5p, miR-182-5p, miR-27a-3p, miR-217, miR-199a-5p, miR-92a-3p, miR-18a-5p, miR-21-5p, miR-221-3p, let-7a-5p, miR-9-5p, miR-137, miR-16-5p, miR-98-5p, miR-30a-5p, miR-122-5p, miR-23a-3p, miR-155-5p, miR-1271-5p, miR-200c-3p, miR-340-3p, miR-29b-3p, miR-211-5p, miR-15b-5p, miR-27b-3p, miR-27b-3p, miR-383-3p/-5p, miR-146a-5p, miR-125b-5p, miR-194-5p, miR-29a-3p, miR-499a-5p, miR-186-5, miR-186-5p, miR-181a-5p, miR-145-5p, miR-15a-5p, miR-21-5p, miR-221-3p, miR-23a-3p, miR-194-5p, and miR-181a-5p, were surveyed (Table 2) [60,61,62,63,64,65,66,67,68,69,70,71,72,73,74,75,76,77,78,79,80,81,82,83,84,85,86,87,88,89,90,91,92,93,94]. Their miRNA changes may be biomarkers for tumor radiation response or function as modulators to regulate radiosensitivity. The impacts of natural-product-regulated miRNAs on the radiation responses of cancer cells are highlighted in the following subsections (Section 3.2.1 and Section 3.2.2).

#### 3.2.1. Some Natural-Product-Regulated miRNAs Can Function as Radiosensitizers in Cancer Cells

Some natural-product-regulated miRNAs exhibit radiosensitizing effects on cancer cells (Table 2) [60,61,62,63,64,65,66,67,68,69,70,71,72,73,74,75,76,77,78,79,80,81,82,83,84,85,86,87,88]. For example, radiation upregulated miR-182-5p expression in T-lymphocyte cultures from healthy donors [178] and head/neck cancer (HNSCC) cells [303]. Overexpressing miR-182-5p improved the radiosensitivity of HNSCC cells by promoting ROS generation [303]. miR-27a-3p mimics enhanced the apoptosis of HNSCC cells. In contrast, miR-27a-3p inhibition improved the proliferation of radiation-treated HNSCC cells [60]. Accordingly, miR-27a-3p promoted radiosensitivity in HNSCC cells. miR-199a-5p enhanced radiosensitivity in breast cancer cells [62], and its radiation target *NUCKS1* was identified (Table 2). miR-92a-3p is targeted by lncRNA HNF1A-AS1, which is overexpressed in lung cancer cells [63]. HNF1A-AS1 knockdown improved radiosensitivity by upregulating miR-92a-3p. Therefore, miR-92a-3p enhanced the radiosensitivity of lung cancer cells [63], and its radiation targets *RNF4*, *MAP3K20*, and *NIPBL* were identified (Table 2).

miR-18a-5p showed radiosensitive effects on lung cancer cells and suppressed lung tumor growth [64], and its radiation targets *ATM*, *RNF4*, and *CCND2* were identified (Table 2). Patients with low miR-21-5p levels showed higher radiosensitivity than those with high miR-21-5p levels. Radiation enhanced let-7a-5p expression in mice [66]. let-7a-5p enhanced radiation-induced tumor repression by inhibiting the self-renewal function of breast cancer stem cells [65], and its radiation targets *CCND2*, *TSPYL5*, and *BCL2L1* were identified (Table 2).

miR-9-5p suppressed the proliferation of nasopharyngeal cancer cells by downregulating hexokinase 2 and enhancing radiosensitivity [67], and its radiation targets *TMEM109* and *MAP3K20* were identified (Table 2). miR-137 is underexpressed in esophageal cancer. The overexpression of miR-137 improved the radiosensitivity of esophageal cancer cells [68], and its radiation targets *MAP3K20*, *RNF4*, *KDM1A*, *NIPBL*, and *NUCKS1* were identified (Table 2). miR-16-5p mimics enhanced the radiosensitivity of cervical [70] and lung [69] cancer cells, and their radiation targets *CCND2*, *YAP1*, and *NUCKS1* were identified (Table 2).

Esophageal-cancer-resistant cells exhibit low levels of miR-98-5p. miR-98-5p mimics enhanced their radiosensitivity, which was reversed by anti-miR-98-5p [71], and their radiation targets *CCND2*, *TSPYL5*, and *BCL2L1* were identified (Table 2). Liver cancer cells exhibit low levels of miR-30a-5p. Radiation or miR-30a-5p mimics showed apoptotic effects on liver cancer cells, whereas a combined treatment (radiation and miR-30a-5p mimic) showed synergistic apoptosis [72], and their radiation target *NUCKS1* was identified (Table 2). Radiation upregulated miR-122-5p in the serum of rectal cancer patients. miR-122-5p mimics enhanced the radiosensitivity of rectal cancer [73], and their radiation target *MAP3K20* was identified (Table 2).

Radiation upregulated miR-155-5p in lung cancer cells [74], suggesting that miR-155-5p may improve radiosensitivity, and its radiation targets *MAP3K20*, *GTF2H5*, *GATA3*, and *MDM2* were identified (Table 2). Liver cancer exhibits a low level of tumor-suppressive miR-1271-5p. The overexpression of miR-1271-5p improved the antiproliferation and radiosensitivity of liver cancer cells [75], and its radiation targets *NIPBL*, *MAP3K20*, and *CCND2* were identified (Table 2). LncRNA PTPRG antisense RNA 1 is upregulated and its target miR-200c-3p downregulated in lung cancer cells. PTPRG overexpression enhanced radioresistance, which was partly alleviated by miR-200c-3p mimics, suggesting that miR-200c-3p can improve radiosensitivity in lung cancer cells [76]. The radiation target of miR-200c-3p, *YAP1*, was identified (Table 2).

Radiation upregulates lncRNA CASC19 and downregulates miR-340-3p in nasopharyngeal cancer cells. LncRNA CASC19 caused the radioresistance of nasopharyngeal cancer cells, which was reversed by miR-340-3p mimics [77]. The radiation target *MDM2* of miR-340-3p was identified (Table 2). miR-29b-3p overexpression reduced the stemness of lung and breast cancer cells in 3D culture, improving their radiosensitivity [78], and its radiation targets *CND2* and *MDM2* were identified (Table 2).

Upregulating miR-211-5p improved the radiosensitivity of rectal cancer by lncRNA EGOT silencing, which was reversed by the miR-211-5p inhibitor [79], and its radiation targets *CCND2*, *NIPBL*, *GTF2H5*, and *EGR1* were identified (Table 2). miR-15b-5p improved the radiosensitivity of colon cancer by upregulation [80], and its radiation targets *CCND2*, *YAP1*, and *NUCKS1* were identified (Table 2). Gastric cancer shows low levels of miR-27b-3p. The overexpression of miR-27b-3p improved radiation-induced antiproliferation and apoptosis, which was reversed by miR-27b-3p knockdown [81], and its radiation targets *SFRP1*, *TLK2*, *GATA3*, and *YAP1* were identified (Table 2).

miR-383-3p and -5p enhanced the apoptosis and UV sensitivity of breast cancer cells [82], and their radiation targets *CRYAB*, *CCND2*, *GATA3*, *NUCKS1*, *MAP3K20*, and *MDM2* were identified, while ATR is targeted by miR-383-5p (Table 2). miR-146a-5p overexpression promoted the apoptosis and radiosensitivity of liver cancer cells [83], and its radiation targets *NUCKS1*, *YAP1*, and *RNF4* were identified (Table 2). miR-125b-5p overexpression improved the radiosensitivity of breast cancer cells [84], and its radiation target *TLK2* was identified (Table 2). miR-29a-3p enhanced the radiosensitivity of oral cancer cells [85], and its radiation targets *CCND2* and *MDM2* were identified (Table 2).

miR-499a-5p overexpression suppressed proliferation and EMT changes and enhanced the radiosensitivity of cervical cancer cells [86], and its radiation target *GATA3* was identified (Table 2). Esophageal cancer shows high levels of circPRKCI and low levels of miR-186-5. CircPRKCI knockdown inhibited the proliferation and enhanced the radiosensitivity of esophageal cancer cells by upregulating miR-186-5p [87], and its radiation targets *YAP1*, *NIPBL*, and *NUCKS1* were identified (Table 2). miR-145-5p overexpression enhanced the radiosensitivity of resistant lung cancer cells [88], and its radiation target *MDM2* was identified (Table 2).

Consequently, these reports suggest that these natural products may have radiosensitizing effects on cancer cells in relation to their radiation-modulating genes predicted based on miRNA.

#### 3.2.2. Some Natural-Product-Regulated miRNAs Can Have a Radioresistance Function in Cancer Cells

Some natural-product-regulated miRNAs have shown radioresistant effects on cancer cells (Table 2) [89,90,91,92,93,94]. For example, radiation exerted different modulating effects on angiogenesis and tumor growth by controlling miR-15a-5p expression [89] (Table 2). High-dose radiation (>10 Gy) decreases miR-15a-5p expression. The miR-15a-5p inhibitor, mimicking high-dose radiation effects, induced antiproliferation, apoptosis, and inflammatory cytokines and suppressed the angiogenesis and tumor growth of a murine colorectal cancer model [89]. Notably, this investigation did not assess the participation of radiation-modulating genes. Utilizing the miRDB [167], the potential radiation-modulating genes of miR-15a-5p, such as *CCND2*, *YAP1*, and *NUCKS1*, were identified (Table 2). This warrants an advanced assessment of the participation of these targets in examining natural-product-associated radiation-modulating effects in connection to miR-15a-5p and its potential targets (*CCND2*, *YAP1*, and *NUCKS1*). circRNA_100367 bound to miR-217 and downregulated the miR-217 of esophageal cancer cells, enhancing radiosensitivity and reducing their survival time [61]. Therefore, miR-217-3p and miR-217-5p enhanced the radioresistance of esophageal cancer cells [61], and their radiation targets *NIPBL* and *ZMPSTE24* were identified (Table 2).

In a cell model, miR-21-5p knockdown enhanced the radiosensitivity of lung cancer cells [90], and its radiation target *YAP1* was identified (Table 2). miR-221-3p is highly expressed in thyroid cancer. The overexpression of miR-221-3p enhanced radioresistance in thyroid cancer cells [91], and its radiation targets *RNF4*, *NIPBL*, and *MDM2* were identified (Table 2). The downregulation of miR-23a-3p could improve the radiosensitivity of oral cancer cells [92], and its radiation targets *MAP3K20*, *EGR1*, and *RNF4* were identified (Table 2). Radiation upregulated miR-194-5p in dying pancreatic-cancer-cell-derived exosomes to promote the proliferation of tumor-repopulating cells [93], suggesting that miR-194-5p may improve the radioresistance of cancer cells. The radiation targets of miR-194-5p, such as *SFRP2*, *YAP1*, and *RNF4*, were identified (Table 2). LncRNA antisense non-coding RNA in the INK4 locus (ANRIL) inhibited the chitooligosaccharide-induced radiosensitivity of colon cancer cells by downregulating miR-181a-5p [94], and its radiation targets *ATM*, *NIPBL*, and *NUCKS1* were identified (Table 2).

Consequently, these reports suggest that these natural products may have radioresistant effects on cancer cells in relation to their radiation-modulating genes predicted based on miRNA.

### 3.3. Function of Natural-Product-Regulated miRNAs in Both Non-Cancer and Cancer Radiation Studies

Some natural-product-regulated miRNAs, such as miR-34a-5p and miR-107, have been included in both non-cancer and cancer radiation studies (Table 2) [7,95,103]. For example, miR-34a-5p was upregulated in mouse liver tissue after whole-body irradiation [95]. EGCG improved the apoptosis and radiosensitivity of liver cancer cells by upregulating miR-34a-5p [7], and its radiation targets *TMEM109* and *GATA3* were identified (Table 2). miR-107 overexpression improved the radiosensitivity of prostate cancer cells [103]. In peripheral mononuclear blood cells, miR-107 was upregulated by radiation [52], and its radiation target *TSPYL5* was identified (Table 2).

In the cases of miR-34a-5p and miR-107, both are upregulated in non-cancer tissues and provide radiosensitivity for cancer cells. This warrants identifying drugs that regulate these miRNAs, which may have functions for radioprotection and radiosensitivity.

### 3.4. Other Natural-Product-Regulated miRNA Candidates May Have Radiomodulating Effects

As mentioned above, most of the natural-product-regulated miRNAs shown in Table 2 are associated with radiation-modulating effects and miRDB results for radiation-modulating targets. However, other natural-product-regulated miRNAs [52,61,68,95,96,97,98,99,100,101,102], such as miR-3960 [95], miR-217-3p [61], miR-22-3p [96,97], miR-126-3p [52], miR-33a-5p [98,99], miR-574-3p [100], miR-370-3p [101], miR-149-5p [102], and miR-451a [52], have only been reported in the context of radiation-modulating effects, without available miRDB results for radiation-modulating targets.

In contrast, several natural-product-regulated miRNAs produced miRDB results for radiation-modulating targets without available reference support. For example, miR-3960 was upregulated by radiation in mouse liver tissue [95]. miR-22-3p overexpression promoted the apoptosis and radiosensitivity of glioma [97] and lung cancer [96]. miR-126-3p was upregulated by radiation in peripheral mononuclear blood cells [52]. miR-33a-5p is present in low levels in melanoma, exhibiting tumor-suppressive effects. miR-33a-5p promoted radiosensitivity by suppressing glycolysis in melanoma, which was reversed by miR-33a-5p knockdown [98]. miR-33a-5p was also upregulated in glioblastoma by radiation [99]. miR-574-3p expression was downregulated by radiation in glioma-patient-derived serum exosomes [100]. circ_NEK6 exhibits high levels of resistance to thyroid cancer. circ_NEK6 knockdown improved the antiproliferation, apoptosis, and radiosensitivity of differentiated thyroid cancer cells by upregulating miR-370-3p [101]. LncRNA opioid growth factor receptor pseudogene 1 (OGFRP1) is highly expressed in gastric cancer tissues and cells. OGFRP1 inhibited the radiosensitivity of gastric cancer by downregulating miR-149-5p [102]. In peripheral mononuclear blood cells, miR-451a was downregulated [52].

Moreover, other natural-product-regulated miRNAs (and targets) shown in Table 2, such as miR-186-3p (*GTF2H5* and *TSPYL5*); miR-204-3p (*MAP3K20*); miR-29c-3p (*CCND2* and *MDM2*); miR 132-3p (*MAP3K20*, *EGR1*, *TSPYL5*, and *NUCKS1*); let-7f-1-3p (*GATA3*, *SFRP2*, *MAP3K20*, and *MDM2*); miR-542-3p (*SFRP1*); miR-590-5p (*YAP1*); miR-215-3p (*TLK2* and *MAP3K20*); miR-215-5p (*GTF2H5* and *NIPBL*); miR-371b-5p (*MDM2* and *KDM1A*); miR-589-5p (*RNF4*); let-7c-5p (*CCND2*, *TSPYL5*, and *BCL2L1*); and miR-10b-5p (*GATA3*), have only been reported in terms of miRDB results for radiation-modulating targets, without available literature reports on their radiation-modulating effects. Accordingly, the potential radiation-modulating effects and targets for these miRNAs warrant further assessment in the future.

Finally, the literature search and miRDB data mining were not possible for radiation-associated miRNAs such as miR-3178, miR-6085, miR-375-5p, miR-181c-3p, miR-129-1-3p, miR-29b-1, miR-99b-3p, miR-549a-5p, miR-1247-5p, and miR-137-5p. The participation of these miRNAs in regulating radiation response may already be under investigation but as yet unreported, or they may show only a weak association with radiation response.

## 4. Connection between Natural-Product-Regulated miRNAs and Exosome Biogenesis-Modulating Effects

The connection between the radiation-modulating effects of natural products (Table 1) and their associated miRNA effects has been explored (Table 2). However, the impacts of these natural-product-regulated miRNAs on exosome biogenesis-modulating functions have not been reported as of yet. A detailed assessment of the relationship between miRNAs and exosome biogenesis-modulating functions is warranted.

Exosome biogenesis, including the processes of secretion and assembly, was investigated using the Gene Ontology function in MGD (GO:1990182) [168]. As exosomal assembly genes, CD34 antigen (*CD34*), syndecan 1 (*SCD1*), *SDC4*, programmed cell death 6 interacting protein (*PDCD6IP*), syndecan binding protein (*SDCBP*), tumor susceptibility gene 101 (*TSG101*), and SH3 domain and ITAM motif (*STAM*) were identified by GO [168]. As exosomal secretion genes, ATPase class II; type 9A (*ATP9A*); *ATP13A2*; COP9 signalosome subunit 5 (*COPS5*); HGF-regulated tyrosine kinase substrate (*HGS*); myosin VB *(MYO5B)*; *PDCD6IP*; RAB7A, a member of the RAS oncogene family (*RAB7A*); *RAB7B*; *RAB11A*; *RAB27A*; parkin RBR E3 ubiquitin-protein ligase (*PRKN, PARK2*); STEAP family member 3 (*STEAP3*); vacuolar protein sorting 4A (*VPS4A*); *VPS4B*; charged multivesicular body protein 2A (*CHMP2A*); *SDC1*; *SDC4*; *SDCBP*; the SNF8 subunit of the endosomal sorting complexes required for transport (ESCRT)-II complex (*SNF8*); sphingomyelin phosphodiesterase 3 neutral (*SMPD3*); *TSG101*; and *STAM* were available in GO [168]. The *PDCD6IP*, *SDC1*, *SDC4*, *SDCBP*, *STAM*, and *TSG101* genes are included in both exosomal assembly and secretion [168].

**Figure 3:**
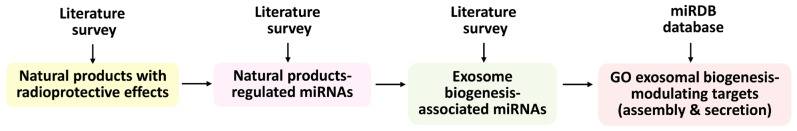
Strategy for filling the knowledge gap regarding the connection between natural products, their associated miRNAs, and exosome biogenesis-modulating targets. Using a PubMed/Google Scholar search, literature surveys for (1) radioprotective natural products and (2) natural-product-regulated miRNAs were performed (Table 1). Next, (3) the impact on exosome biogenesis of these miRNAs was assessed in both non-cancer and cancer exosomes by a literature search. Finally, (4) these miRNAs were fed into miRDB [167] to retrieve the exosome biogenesis-modulating genes summarized from the Gene Ontology function in MGD (GO:1990182) [168].

Following the strategy outlined in Figure 3, several miRNAs associated with radiation-modulating natural products and their potential connections to exosome biogenesis-modulating effects and target genes were assessed. Although the natural products were radioprotectors (Table 1; step 1), their modulated miRNAs (Table 1; step 2) were retrieved from studies unrelated to exosome biogenesis. A literature search was performed to test the potential effects of modifying these miRNAs on exosome biogenesis (step 3). Notably, miRNA candidates could impact exosome biogenesis for both non-cancer and cancer cells. Finally, exosome biogenesis-associated miRNAs were fed into miRDB to predict GO exosome biogenesis-modulating targets (step 4).

After the literature search (Figure 3), we identified some miRNAs that were addressed in non-cancer radiation studies (Section 4.1), while others were associated with cancer radiation studies (Section 4.2). Notably, some miRNAs included in non-cancer exosome studies were also reported in cancer exosome studies and vice versa (Section 4.3). However, this review cannot list them all, though some are described below (Table 3).

### 4.1. Function of Natural-Product-Regulated miRNAs in Non-Cancer Exosome Studies

The potential exosome biogenesis-modulating genes were retrieved from the target search results by inputting natural-product-modulating miRNAs [104,105,106,107,108,109,110,111,112,113,114]. Consequently, the potential exosome biogenesis-modulating gene targets for the natural-product-modulating miRNAs were individually identified. The bioinformatic target prediction of exosome biogenesis genes for each natural-product-modulating miRNA was performed using miRDB [167]. For non-cancer radiation studies, several natural-product-regulated miRNAs and their potential exosome biogenesis-modulating targets are described below. All the potential exosome biogenesis-modulating targets identified using miRDB are referred to as exosome targets.

For example, exosomal miR-199a-5p induced hepatic lipid accumulation in mice [105] and suppressed the apoptosis and inflammation of neural cells [104], and its exosome targets *ATP13A2*, *RAB7A*, and *ATP9A* were identified (Table 3). Senescent HUVEC-cell-derived exosomes were rich in miR-21-5p, improving the delivery of senescence signals to inhibit proliferation [106], and its exosome targets *RAB11A* and *MYO5B* were identified (Table 3). Exosomal let-7a-5p derived from osteoclasts enhanced the differentiation of chondrocyte hypertrophy [107], and its exosome targets *STEAP3* and *MYO5B* were identified (Table 3). Bone-marrow-derived mesenchymal stem cells coud generate exosomal miR-9-5p to reduce osteoarthritis [108], and its exosome targets *STEAP3*, *PDCD6IP*, *STAM*, *SDC1*, *SMPD3*, and *CD34* were identified (Table 3).

High levels of exosomal miR-30a-5p derived from vascular endothelial cells suppressed the proliferation and migration of lung cancer cells [109], and its exosome target *RAB11A* was identified (Table 3). M2-macrophage-derived exosomal miR-1271-5p suppressed the apoptosis of hypoxia-induced cardiomyocytes and caused cardiac damage in mice with acute myocardial infarction [110], and its exosome targets *RAB27A*, *RAB7A*, and *MYO5B* were identified (Table 3). Exosomal miR-29b-3p was highly expressed in the bone marrow mesenchymal stem cells of aged mice [111]. Overexpressing this exosomal miR-29b-3p promoted insulin resistance in young mice, while inhibiting exosomal miR-29b-3p suppressed insulin resistance in aged mice [111]. The exosome target of miR-29b-3p, *SMPD3*, was identified (Table 3).

Salivary exosomal miR-223-3p in periodontitis was lower than in healthy controls. miR-223-3p knockdown upregulated pyroptosis [112], and its exosome targets *MYO5B* and *STAM* were identified (Table 3). Under hypoxia, exosomal miR-27b-3p was overexpressed in cardiac microvascular endothelial cells, which was reversed by miR-27b-3p inhibition [129], and its exosome target *SMPD3* was identified (Table 3). In an intracerebral hemorrhage, exosomal miR-383-3p in activated microglia induced the necroptosis of neurons [113], and its exosome targets *SMPD3*, *TSG101*, and *SDCBP* were identified (Table 3). Exosome miR-371b-5p stimulated the proliferation but not the differentiation of lung alveolar progenitor type II cells [114], and its exosome targets *RAB11A*, *STAM*, and *SDCBP* were identified (Table 3).

Consequently, these reports suggest that these natural products may have exosome biogenesis-modulating effects on non-cancer exosomes in related to their exosome biogenesis-modulating genes predicted based on miRNA.

### 4.2. Function of Natural-Product-Regulated miRNAs in Cancer Exosome Studies

Several natural-product-regulated miRNAs modulating cancer exosomes are described below (Table 3) [93,106,115,116,117,118,119,120,121,122,123,124,125,126,127,128,129,130,131,132,133,134,135,136,137,138,139,140,141,142]. miR-15a-5p was overexpressed in cancerous exosomes to inhibit liver cancer cell proliferation [115], and its exosome targets *MYO5B* and *VPS4A* were identified (Table 3). Hypoxic glioblastoma cells generated more exosomes and a higher miR-182-5p content in exosomes than those in a normoxic state, improving angiogenesis, which was reversed by miR-182-5p knockdown [116], and its exosome targets *RAB7A*, *ATP9A*, and *SDC1* were identified (Table 3). Liver cancer tissues and cell lines showed a lower level of miR-27a-3p than non-cancer controls. Exosomal miR-27a-3p derived from mesenchymal stem cells inhibited the proliferation and metastasis of liver cancer cells [117], and its exosome target *SMPD3* was identified (Table 3).

Exosomal miR-92a-3p was rich in liver cancer tissues [119] and serum from gastric cancer patients [118], and its exosome target *VPS4B* was identified (Table 3). Exosomal miR-221-3p derived from cervical cancer cells enhanced lymphangiogenesis and metastasis in lymph nodes [120], and its exosome target *PDCD6IP* was identified (Table 3). miR-34a-5p overexpression in exosomes derived from cancer-associated fibroblasts showed inhibitory effects on the proliferation and metastasis of oral cancer cells [121], and its exosome target *VPS4A* was identified (Table 3). Serum exosomal miR-16-5p inhibited proliferation and triggered the apoptosis of lung cancer cells [304], and its exosome targets *MYO5B* and *VPS4A* were identified (Table 3).

Patients with high-risk neuroblastoma showed a low level of miR-186-3p. Exosomal miR-186 derived from natural killer cells suppressed neuroblastoma growth [123], and its exosome target *VPS4B* was identified (Table 3). Serum exosomes of glioblastoma patients exhibited high levels of miR-98-5p [124], and its exosome targets *STEAP3* and *MYO5B* were identified (Table 3). Nasopharyngeal cancer-cell-derived exosomal miR-17-5p enhanced proliferation and angiogenesis [125], and its exosome targets *TSG101* and *MYO5B* were identified (Table 3). The exosomal miR-200-3p family (miR-141-3p, miR-200a-3p, miR-200b-3p, and miR-200c-3p), particularly miR-200c-3p, functioned as a promising diagnostic serum marker for cholangiocarcinoma [126]. The exosome targets of miR-200c-3p (*PRKN* and *STAM*) were identified (Table 3).

Omental cancer-associated fibroblasts showed low levels of exosomal miR-29c-3p, improving the peritoneal metastasis of ovarian cancer cells; this was reversed by overexpressing miR-29c-3p [127], whose exosome target *SMPD3* was identified (Table 3). Overexpressing exosomal miR-15b-5p from laryngeal cancer enhanced proliferation [128], and its exosome targets *MYO5B* and *VPS4A* were identified (Table 3). Exosomal miR-19b-3p was highly expressed in the serum of glioblastoma patients [124], and its exosome targets *SDC1*, *VPS4B*, and *MYO5B* were identified (Table 3).

Exosomal miR-107 was highly expressed in gastric cancer cells [130], and its exosome targets *VPS4A* and *SDCBP* were identified (Table 3). Exosomal miR-125b-5p enhanced the migration and EMT of pancreatic cancer cells, with the degree of metastasis proportional to the miR-125b-5p level [131], and its exosome target *VPS4B* was identified (Table 3). Exosomal miR-590-5p was overexpressed in the serum of gastric cancer patients [132]. High exosomal miR-590-5p suppressed the proliferation and migration of gastric cancer cells, and its exosome targets *RAB11A* and *MYO5B* were identified (Table 3).

The serum of breast cancer patients exhibited high levels of exosomal miR-370-3p, which was associated with tumor proliferation, migration, and stemness progression [133], and its exosome targets *ATP9A*, *RAB11A*, and *RAB7A* were identified (Table 3). Exosomal miR-194-5p derived from dying pancreatic cancer induced DNA damage response in tumor-repopulating cells to promote tumor repopulation [93], and its exosome target *SDC4* was identified (Table 3). Hypoxia downregulated exosomal miR-29a-3p, while the upregulation of miR-29a-3p inhibited proliferation and triggered apoptosis in glioma cells [134], and its exosome target *SMPD3* was identified (Table 3). Overexpressing exosomal miR-149-5p suppressed the metastasis and growth of pituitary tumors [135], and its exosome targets *VPS4A* and *CD34* were identified (Table 3).

Exosomal miR-499a-5p in highly metastatic lung cancer cells enhanced cell proliferation and migration, which was reversed by the miR-499a-5p inhibitor [136], and its exosome target *ATP9A* was identified (Table 3). Exosomal miR-186-5p in bladder cancer cells promoted natural killer (NK) cell dysfunction and suppressed the cell-killing effects of NK cells [137], and its exosome targets *VPS4B*, *ATP9A*, *RAB27A*, and *STAM* were identified (Table 3). Overexpressing exosomal miR-34c-5p reduced the radioresistance of nasopharyngeal cancer cells [138], and its exosome target *VPS4A* was identified (Table 3).

Osteosarcoma tissues and exosomes show high levels of miR-181a-5p. Osteosarcoma-cell-derived exosomal miR-181a-5p enhanced macrophage M2 polarization [139], and its exosome targets *PDCD6IP* and *PRKN* were identified (Table 3). Higher stages of ovarian cancer exhibit a low level of miR-145-5p. The downregulation of exosomal miR-145-5p in ovarian cancer cells improved ovarian cancer development [140], and its exosome target *STAM* was identified (Table 3). Exosomal let7c-5p inhibited the proliferation and migration of breast cancer cells [141], and its exosome targets *STEAP3* and *MYO5B* were identified (Table 3). Serum exosomal miR-10b-5p is highly expressed in liver cancer patients, particularly in the early stage. High serum exosomal miR-215-5p showed low disease-free survival in liver cancer patients [142], and its exosome targets *SDC1* and *SMPD3* were identified (Table 3).

Consequently, these reports suggest that these natural products may have exosome biogenesis-modulating effects on cancer exosomes in relation to their exosome biogenesis-modulating genes predicted based on miRNA.

### 4.3. Other Natural-Product-Regulated miRNAs May Have Exosome Biogenesis-Modulating Effects

As mentioned above, most radiation-modulating natural-product-regulated miRNAs (Table 3) are associated with exosome biogenesis and miRDB results for exosome biogenesis-modulating targets. However, other miRNAs [143,144,145,146,147,148,149,150,151,152,153,154,155,156,157,158,159,160,161,162,163,164,165,166], such as miR-3960 [143], miR-6085 [144], miR-18a-5p [145], miR-22-3p [146], miR-126-3p [147], miR-137-3p [148], miR-122-5p [149], miR-23a-3p [150], miR-155-5p [151], miR-33a-5p [152], miR-574-3p [153], miR-132-3p [154], miR-211-5p [155], miR-375-3p [156], miR-181c-3p [157], miR-146a-5p [158], miR-542-3p [159], miR-877-3p [160], miR-192-5p [161], miR-451a [162], miR-215-5p [163], miR-99b-3p [164], miR-549a-5p [165], and miR-1247-5p [166], have only been reported in terms of exosome biogenesis-modulating effects, without available miRDB results for exosome biogenesis-modulating targets.

In contrast, several natural-product-regulated miRNAs produced miRDB results for radiation-modulating targets without available literature reports. For example, the overexpression of miR-3960 suppressed the proliferation-promoting effects of pancreatic-cancer-cell-derived exosomes [143]. M2-macrophage-derived exosomes contain a higher miR-6085 content than M0-exosomes, improving the osteogenic differentiation of periodontal ligament stem cells [144]. The inhibition of exosomal miR-18a-5p alleviated the metastasis-promoted osteoblastic damage [145] that commonly occurs in prostate cancer [305]. Colon cancer tissues and cells contain a low level of miR-22-3p. Exosomes derived from senescent human umbilical vein endothelial cells (HUVECs) contain higher miR-217-3p levels, transferring senescence signals to inhibit proliferation [106]. Mesenchymal stem-cell-derived exosomal miR-22-3p inhibited the proliferation and invasion of colon cancer cells [146]. Exosomal miR-126-3p derived from bone marrow mesenchymal stem cells suppressed lung cancer cell proliferation and triggered apoptosis in vitro and in vivo [147].

Patients with Parkinson’s disease (PD) exhibited high serum exosomal miR-137-3p. The knockdown of miR-137-3p reduced the PD-induced oxidative stress injury of neurons [148].

Gastric cancer patients exhibited a low level of serum exosomal miR-122-5p [149]. Exosomal miR-122-5p suppressed the proliferation and metastasis of gastric cancer cells. miR-23a-3p is overexpressed in cholangiocarcinoma. Exosomal miR-23a-3p promoted the proliferation and metastasis of cholangiocarcinoma, which was reversed by miR-23a-3p knockdown [150]. miR-155-5p is highly expressed in gastric cancer tissues and cells. Exosomal miR-155-5p is rich in gastric cancer cells and enhanced cell proliferation and migration [151]. miR-33a-5p was underexpressed in oxaliplatin-resistant colon cancer cells compared to sensitive control cells. Exosomal miR-33a-5p was present at only a low level in resistant colon cancer cells [152]. Placenta-derived exosomal miR-574-3p was underexpressed after chemerin, enhancing proliferation and angiogenesis for gestational diabetes mellitus [153]. Mesenchymal stem-cell-derived exosomal miR-132-3p improved proliferation, inhibited apoptosis in an in vitro inflammatory cell model, and reduced LPS-induced acute lung injury in mice [154]. Exosomal miR-211-5p in highly metastatic melanoma cells could enhance the metastatic function of weakly metastatic melanoma cells [155].

Exosomal miR-375-3p enhanced vascular barrier permeability and lung cancer metastasis [156]. Exosomal miR-181c-3p in cortical neurons showed anti-neuroinflammatory effects in rat astrocytes [157]. Advanced lung cancer patients exhibited low levels of exosomal miR-146a-5p in the serum, accompanied by high recurrence compared to patients with high levels [158]. Exosomal miR-542-3p in bone marrow mesenchymal stem cells enhanced mouse wound repair [159]. Exosomal miR-877-3p was highly expressed in the urine of patients with diabetic kidney disease [160]. Exosomal miR-192-5p was downregulated in the plasma of epithelial ovarian cancer patients [161]. Exosomal miR-451a triggered apoptosis, inhibiting migration and angiogenesis in liver cancer cells [162]. Adipose-derived stem cells exhibited a high level of exosomal miR-215-5p, suppressing the EMT of podocytes [163]. Exosomal miR-99b-3p derived from mesenchymal stem cells suppressed microglial activation by enhancing autophagy [164]. Exosomal miR-549a was underexpressed in tyrosine kinase inhibitor (TKI)-resistant renal cancer cells and their exosomes. Low-level exosomal miR-549a improved angiogenesis and metastasis in TKI-resistant renal cancer cells [165]. Myelodysplastic syndrome patients exhibited ineffective hematopoiesis and displayed a high risk of acute myeloid leukemia. Exosomal miR-1247-5p showed high levels in MDS plasma [306].

Moreover, other natural-product-regulated miRNAs shown in Table 3, such as miR-217-5p (*ATP9A*, *STEAP3*, and *PDCD6IP*); miR-204-3p (*RAB11A*); miR-340-3p (*RAB11A*); let-7f-1-3p (*SDCBP* and *RAB7A*); miR-147a (*ATP9A*); miR-29b-1 (*COPS5*); and miR-124-5p (*PDCD6IP*), provided miRDB results for exosome biogenesis-modulating targets without available literature reports on their exosome biogenesis-modulating effects. Accordingly, the potential exosome biogenesis-modulating effects and targets for these miRNAs warrant further assessment in the future.

Finally, the literature search and GO miRDB data mining were not possible for radiation-associated miRNAs such as miR-3178, miR-137-5p, miR-518b, miR-375-5p, miR-383-5p, miR-129-1-3p, miR-215-3p, miR-589-5p, and miR-137-5p. It is possible that the participation of these natural-product-regulated miRNAs in regulating radiation response is still under investigation, or that they show a weak association with radiation response.

## 5. Relationship between Radiation and Exosome Biogenesis Modulation by Natural-Product-Regulated miRNAs

Among the 80 miRNA candidates (Table 2 and Table 3), 61 miRNAs overlapped in terms of radiation- and exosome biogenesis-modulating effects, including let-7a-5p, let-7c-5p, let-7f-1-3p, miR-107, miR-10b-5p, miR-122-5p, miR-124-5p, miR-125b-5p, miR-1271-5p, miR-132-3p, miR-137-3p, miR-145-5p, miR-146a-5p, miR-147a, miR-149-5p, miR-155-5p, miR-15a-5p, miR-15b-5p, miR-16-5p, miR-181a-5p, miR-182-5p, miR-186-3p, miR-186-5p, miR-18a-5p, miR-192-5p, miR-194-5p, miR-199a-5p, miR-19b-3p, miR-200c-3p, miR-204-3p, miR-211-5p, miR-215-5p, miR-21-5p, miR-217-3p, miR-217-5p, miR-221-3p, miR-223-3p, miR-22-3p, miR-23a-3p, miR-27a-3p, miR-27b-3p, miR-29b-3p, miR-29c-3p, miR-30a-5p, miR-33a-5p, miR-340-3p, miR-34a-5p, miR-34c-5p, miR-370-3p, miR-371b-5p, miR-375-3p, miR-383-3p, miR-3960, miR-451a, miR-499a-5p, miR-542-3p, miR-574-3p, miR-590-5p, miR-877-3p, miR-9-5p, and miR-98-5p. These results indicated that the same natural-product-modulating miRNAs may exert bifunctional roles in regulating radiation- and exosome biogenesis-modulating effects. However, the potential interaction between the radiation and exosome biogenesis modulation by natural-product-regulated miRNAs remains unclear.

**Figure 4:**
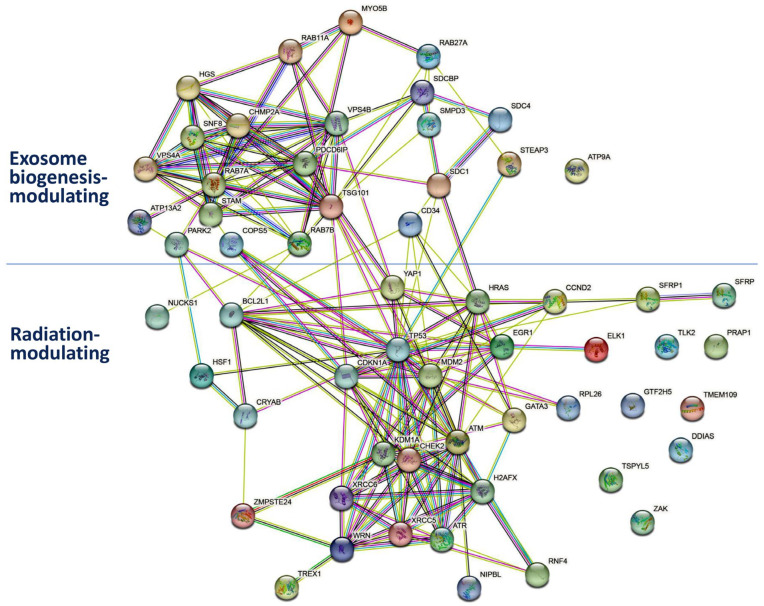
Interaction analysis for radiation- and exosome biogenesis-modulating targets. These targets, identified by Gene Ontology (GO:0071480, GO:0071481, and GO:1990182) [168], are listed in Table 2 and Table 3. The potential protein–protein interactions of these targets was analyzed using the STRING database [307]. Radiation- and exosome biogenesis-modulating targets are grouped into top and bottom parts of the figure, respectively. The connecting lines indicate potential protein–protein interactions between the proteins at the ends of each line. Proteins with no recorded interactions are shown on the right side without connecting lines.

To evaluate the potential interaction between radiation and exosome biogenesis targets, a protein–protein interaction analysis using the STRING database was conducted (Figure 4). Most GO-provided targets showed interaction within the same function for radiation or exosome biogenesis modulation. The analysis showed the complex interactions between targets of the radiation-modulating function. Similar interactions were demonstrated for the exosome biogenesis function. In addition to self-interaction for radiation- and exosome biogenesis-modulating targets, some exosome biogenesis-modulating targets could interact with radiation-modulating targets.

For example, the exosome biogenesis target PARK2 may interact with the radiation-modulating targets HSF1, BCL2L1, and TP53. The exosome biogenesis target COPS5 may interact with the radiation-modulating targets CDKNIA, TP53, and ATM. The exosome biogenesis target TSG101 may interact with the radiation-modulating targets CDKNIA, TP53, MDM2. The exosome biogenesis target VPS4B may interact with the radiation-modulating target YAP1. The exosome biogenesis target SDC1 may interact with the radiation-modulating targets TP53 and HRAS. The exosome biogenesis target RAB7B may interact with the radiation-modulating targets NUCKS1 and BCL2L1. The exosome biogenesis target CD34 may interact with the radiation-modulating targets BCL2L1, TP53, MDM2, GATA3, and HRAS. Therefore, there may be an interplay between radiation-modulating targets and exosome biogenesis targets.

Collectively, natural-product-regulated miRNAs may control the expression of radiation- and exosome biogenesis-modulating targets at the transcriptional level, while radiation- and exosome biogenesis-modulating targets may participate in subtle protein–protein interactions to regulate natural-product-mediated radiomodulation and exosome biogenesis. This warrants a detailed assessment of the interaction between radiation and exosome biogenesis with experimental validation for natural product treatments in the future.

## 6. Overview of Natural Products That Regulate miRNAs to Modulate Radiation Responses

The relationship between natural products, their modulated miRNAs, and their potential radiation-modulating targets identified from mining miRDB was described with a focus on natural products and miRNAs (Table 1, Table 2 and Table 3). To address the final gene targeting, this miRNA–radiation-target axis was plotted by converging to their target genes (Table 4).

Different natural-product-regulated miRNAs may target the same radiation-modulating genes (Figure 3). For example, *ATM* is targeted by miR-18a-5p and miR-181a-5p. *BCL2L1* is targeted by let-7a-5p, let-7c-5p, and miR-98-5p. A similar target–miRNA relationship is shown in Figure 3, but this is not described due to the large number of miRNAs involved.

Some natural-product-regulated miRNAs potentially target different radiation-modulating genes. For example, let-7a-5p targets *BCL2L1*, *CCND2*, and *TSPYL5* (Table 4). miR-27a-3p targets *GATA3*, *SFRP1*, *TLK2*, and *YAP1*. miR-155-5p targets *GTF2H5*, *MAP3K20*, and *MDM2*. miR-17-5p targets *CCND2*, *DDIAS*, and *MAP3K20*. In comparison, some natural-product-regulated miRNAs mainly target specific genes. miR-21-5p, targeting *YAP1* alone, is associated with different natural products, such as betulinic acid, ligustrazine, genistein, lycopene, and withaferin A.

Some natural products can potentially modulate miRNAs and their radiation-modulating targets identified by mining miRDB (Table 4). For example, withaferin A shows the potential to regulate let-5a-5p/let-5c-5p to target *BCL2L1*, *CCND2*, and *TSPYL5*; miR-124-5p to target *DDIAS* and *TLK2*; miR-10b-5p to target *GATA3*; and miR-27a-3p to target *GATA3*, *SFRP1*, *TLK2*, and *YAP1*. Fucoidan can potentially regulate miR-17-5p to target *CCND2*, *DDIAS*, and *MAP3K20*, and miR-29b-3p/miR-29c-3p to target *CCND2* and *MDM2*. Ursolic acid shows the potential to regulate miR-34c-5p to target *GATA3* and *TMEM109*; miR-186-5p to target *NIPBL*, *NUCKS1*, and *YAP1*; miR-499a-5p to target *GATA3*; and miR-34a-5p to target *TMEM109*. Piperine shows the potential to regulate miR-383-5p to target *ATR*; miR-383-3p to target *CCND2*, *GATA3*, *MAP3K20*, *NUCKS1*, and *CRYAB*; and miR-17-5p to target *CCND2* and *DDIAS*. Many natural products showing differential regulation to target radiation-modulating genes are not comprehensively described here due to the difficulty of succinctly illustrating all relationships (Table 4).

Consequently, the axis of natural products, miRNAs, and radiation-modulating targets is presented in Table 4.

## 7. Overview of Natural Products That Regulate Exosomal miRNAs Modulating Exosome Biogenesis

The relationship between natural products, their natural-product-modulated exosomal miRNAs, and potential exosome biogenesis-modulating targets was presented with a focus on miRNAs in Table 1, Table 2 and Table 3. To address the final gene targeting, this natural-product–miRNA–exosome biogenesis target axis is illustrated by referring to their target genes in Table 5.

Different miRNAs can target the same exosome biogenesis-modulating genes (Table 5). For example, *PDCD6IP* is targeted by miR-217-5p, miR-221-3p, miR-9-5p, miR-181a-5p, and miR-124-5p. *SDCBP* is targeted by let-7f-1-3p, miR-107, miR-383-3p, and miR-371b-5p. A similar target–miRNA relationship is shown in Table 4 but is not described here due to there being too many available miRNAs.

Some miRNAs potentially target different exosome biogenesis-modulating genes. For example, let-7a-5p targets *MYO5B* and *STEAP3* (Table 5), miR-21a-5p targets *MYO5B* and *RAB11A*, miR-17-5p targets *MYO5B* and *TSG101*, miR-182-5p targets *RAB7A* and *SDC1*, miR-200-3p targets *PRKN* and *STAM*, and miR-15a-5p/miR-16-5p targets *MYO5B* and *VPS4A*. In comparison, some miRNAs mainly target specific genes. miR-21-5p, targeting *MYO5B* and *RAB11A* alone, is associated with different natural products, such as diosmin, gallic acid, ginsenoside Rg1, hesperidin, matrine, quercetin, and chrysin.

Some natural products can modulate miRNAs and their exosome biogenesis-modulating targets (Table 5). For example, withaferin A shows the potential to regulate let-5a-5p/let-5c-5p to target *MYO5B* and *STEAP3*, miR-124-5p to target *PDCD6IP*, miR-10b-5p to target *SDC1* and *SMPD3*, and miR-27a-3p to target *SMPD3*. Fucoidan can potentially regulate miR-17-5p to target *MYO5B* and *TSG101*, and miR-29b-3p/miR-29c-3p to target *SMPD3*. Ursolic acid can potentially regulate miR-186-5p to target *ATP9A*, *RAB27A*, *STAM*, and *VPS4B*, and miR-149-5p to target *CD34*. Piperine can potentially regulate miR-383-3p to target *SDCBP*, *SMPD3*, and *TSG101*, and miR-17-5p to target *CMYO5B* and *TSG101*. Several natural products show differential regulation to target exosome biogenesis-modulating genes. However, these have not been sufficiently described, or the available information is too scattered to present all relationships in appropriate detail (Table 5).

This review summarizes and evaluates the axis of natural products, miRNAs, and exosome biogenesis targets in Table 5.

## 8. Conclusions

Radiotherapy is effective in cancer treatments but is limited by its adverse side effects, particularly for non-cancer tissue and cell injury. Several radioprotectors have been developed, but natural products exhibiting less toxicity than chemical compounds are preferable radioprotectors. Although several radioprotective natural products have been reported, the potential radiomodulating mechanisms remain unclear, particularly for radiation- and exosome biogenesis-modulating signaling and miRNA-associated responses.

Radiation and natural products can modulate miRNAs and exosome biogenesis. That being said, there are some knowledge gaps related to the connections between radiomodulating natural products and miRNAs and between these miRNAs and target radiation- and exosome biogenesis-modulating genes. Introducing the bioinformatic tools miRDB and the GO database allowed us to retrieve the potential targets of miRNAs associated with radiomodulating natural products.

**Figure 5:**
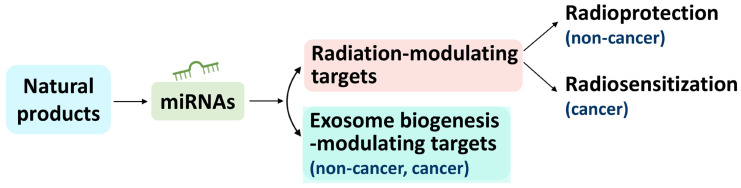
Schematic summary. Many radioprotective natural products were searched for in the literature. Using these natural product candidates, their miRNA changes were retrieved. In view of the miRNAs, the potential targets for modulating radiation and exosome biogenesis were predicted by the bioinformatic tool miRDB. Literature reports also demonstrate that these miRNAs can regulate responses to radiation and exosome biogenesis. Interestingly, radioprotective natural products may modulate several miRNAs, and, in turn, miRNAs exert radioprotection/radiosensitization and exosome biogenesis in terms of miRDB target prediction. Some natural products may have radioprotective and radiosensitizing effects. Detailed investigations into the radiomodulation of natural products are warranted. Moreover, most of the abovementioned miRNAs are bifunctional for radiation and exosome biogenesis modulation, suggesting that their interaction may modulate the radiomodulation effects of natural products.

In the present review, we proposed a strategy to identify radioprotective natural products, find the miRNA candidates of these natural products, and start surveying the potential targets of radiation- and exosome biogenesis-modulating genes based on these miRNAs. According to the literature survey, these miRNA candidates were found to be responsive to radiation in non-cancer and/or cancer tissues. Some miRNAs showed radioprotective effects on non-cancer tissues, and some showed radiosensitive or radioresistant effects on cancer tissues (Figure 5). Moreover, most of these targets for modulating radiation response and exosome biogenesis have rarely been investigated, providing a future direction to advanced the study of radiomodulative natural products.

A concern regarding radioprotectors for non-cancer tissues and cells is the unplanned protection of tumor tissues and cells from being killed by radiotherapy, thus leading to radioresistance [5]. In Table 1, apigenin, baicalein, CAPE, chrysin, curcumin, daidzein, EGCG, gallic acid, genistein, quercetin, resveratrol, silymarin, vitamin C, and zingerone were not reported to exhibit radioprotective effects on tumor cells [5]. In addition to radioprotector function (Table 1), some natural products, such as curcumin, emodin, genistein, resveratrol, berberine, celastrol, ursolic acid, vitamin D, withaferin A [308], EGCG, CAPE, quercetin, and fucoidan [4], have also been reported to exhibit radiosensitizing effects on cancer cells. Accordingly, these natural products show dual functions by improving radiosensitization [4,308] in cancer cells and radioprotection (Table 1) in non-cancer cells. Except for those mentioned above, the potential radiosensitive effects of the remaining radioprotectors (Table 1) were outside the scope of this review, because the present review mainly focused on radioprotective natural products.

Moreover, this review considered highly active substances of plant origin from terrestrial biota in great detail as radioprotectors. Some bioactive substances isolated from marine biota, such as algae and invertebrates of the world’s oceans, were also described. Almost all isolated producers of marine biota have very promising anti-cancer activity. Marine biota are quite easy to grow on marine farms; therefore, the future of pharmacy combatting cancer lies in these active ingredients. A detailed investigation of radioprotectors derived from marine natural products is warranted in the future.

Notably, the resources of the miRDB targets for radiation- and exosome biogenesis-modulating effects were derived from different cell types, which may incur different targets for various miRNAs. Notably, these potential targets for miRNAs were the predicted results of the miRDB and still need experimental validation. This review cannot exclude the possibility that natural products may regulate other functional miRNAs to modulate radiation and exosome biogenesis besides those mentioned here. The proposed rationale for the natural-product–miRNA–downstream axis still warrants a detailed illustration. The potential interaction between radiation and exosome biogenesis in natural product treatments needs further assessment using inhibitors against radiation and exosome biogenesis targets. Collectively, thoughtful investigation is required to validate the detailed changes in miRNAs and the potential targets for regulating radiomodulation and exosome biogenesis within radiation studies using natural products for wet experiments in the future.

In conclusion, this review presented well-organized connections between natural products, miRNAs, radiomodulation, and exosome biogenesis (Figure 5), providing directions for future investigations into natural-product-based radiotherapy through the modulation of radiation- and exosome-biogenesis.

## Figures and Tables

**Table 1:** Radioprotective natural products and their regulated miRNAs.

Radioprotective Natural Product	Natural-Product-Regulated miRNAs	Radioprotective Natural Product	Natural-Product-Regulated miRNAs
Apigenin [169]	miR-15a-5p [170]	Withaferin A [16]	let-7c-5p, let-7a-5p [171]
Baicalein [10]	miR-3178 [172]		miR-549a-5p, miR-1247-5p, miR-124-5p, miR-137-5p [171], miR-10b-5p, miR-27a-3p [173] ↓
Celastrol [12]	miR-6085 [174] ↓	Chrysin [175]	miR-92a-3p [176], let-7a-5p, miR-9-5p, miR-22-3p [177], miR-34a-5p [177,178], miR-126-3p [177]
Chlorogenic acid [179]	miR-199a-5p [180]		miR-18a-5p [177], miR-21-5p [181,182], miR-221-3p [177] ↓
Daidzein [5]	miR-122-5p [183]		
Diosmin [184]	miR-21-5p [181,182]	Delphinidin [185]	miR-23a-3p [186], miR-204-3p [187]
	miR-155-5p [181], miR-17-5p [188] ↓	Epigallocatechin gallate (EGCG) [189]	miR-30a-5p [190]
Melatonin [191]	miR-155-5p [192], miR-23a-3p [193] ↓		
Silymarin [5]	miR-122-5p, miR-192-5p, miR-194-5p [194]	Ferulic acid [195]	miR-200c-3p [196]
			miR-340-3p [197] ↓
Thymol [198]	miR-29a-3p [199]	Ginsenoside Rg1 [17]	miR-126-3p [200]
Troxerutin [201]	miR-146a-5p [202], miR-147a [203]		miR-21-5p [181,182] ↓
Vitamin C [204]	miR-215-3p, miR-215-5p, miR-371b-5p, miR-181a-5p [205]	Ligustrazine [206]	miR-211-5p [207], miR-27a-3p [208]
			miR-16-5p [209] ↓
	miR-29b-1, miR-589-5p, miR-451a [210] ↓	Lycopene [211]	let-7f-1-3p [212]
			miR-27a-3p [213] ↓
Zingerone [214]	miR-200c-3p [215]	Piperine [216]	miR-181c-3p [217]
Berberine [9]	miR-182-5p [218]		miR-17-5p [219], miR-383-3p, miR-383-5p [220] ↓
Betulinic acid [221]	miR-27a-3p [222]	Resveratrol [223]	miR-34a-5p [224], miR-542-3p, miR-125b-5p [225] ↓
		Vitamin D [226]	miR-145-5p [227], miR-99b-3p [228], miR-15a-5p [170,228,229]
CAPE [230]	miR-3960 [231]		
Carvacrol [198]	miR-217-3p, miR-217-5p [232]	Esculentoside A [233]	
Curcumin [234]	miR-137-3p, miR-137-5p [235], miR-16-5p [236], miR-98-5p [237], miR-30a-5p [238]		
		Allicin [239]	
	miR-186-3p [240] ↓	Caffeine [241]	
3,3′-Diindolylmethane [242]	miR-21-5p [181,182]	Chlorophyllin [243]	
Emodin [15]	miR-1271-5p [244]	Dehydrozingerone [8]	
Fucoidan [245]	miR-29b-3p [246], miR-29c-3p [247]	*N*-Acetyl tryptophan glucopyranoside [248]	
	miR-17-5p [249] ↓		
Gallic acid [250]	miR-182-5p [251], miR-518b [252]	Gymnemagenin [253]	
	miR-21-5p [181,182] ↓		
Genistein [254]	miR-574-3p [255]	Psoralidin [256]	
	miR-27a-3p [257], miR-155-5p [258], miR-223-3p [259] ↓	Quinic acid [179]	
Hesperidin [260]	miR-16-5p, miR-34a-5p [261], miR-132-3p [262]	Vanillin [263]	
		Zymosan A [264]	
	miR-21-5p [181,182] ↓	Dieckol [265]	
Mangiferin [11]	miR-15b-5p [266], miR-27b-3p, miR-92a-3p [267]	Eckol [268]	
Matrine [269]	miR-22-3p [177], miR-19b-3p [270]	Phloroglucinol [271]	
	miR-21-5p [181,182] ↓		
Parthenolide [272]	miR-107 [273]	Triphlorethol-A [274]	
	miR-375-3p, miR-375-5p [275] ↓		
Quercetin [276]	let-7a-5p [277], miR-146a-5p [277]	Diphlorethohydroxycarmalol [278]	
	miR-21-5p [181,182] ↓		
Rutin [279]	miR-590-5p [280], miR-877-3p [281], miR-129-1-3p [282]		
		Laminaran [283]	
Sesamol [284]	miR-370-3p [285]	β-carotene [286]	
Ursolic acid [287]	miR-149-5p [288], miR-186-5p [289], miR-34c-5p [290]	Astaxanthin [291]	
	miR-499a-5p [292] ↓	Cumaside [293]	

↓ indicates that miRNAs were downregulated by natural products, while miRNAs without ↓ indicate upregulation by natural products.

**Table 2:** The radiation-modulating effects and targets of natural-product-regulated miRNAs.

Natural-Product-Regulated miRNA	Radiation-Associated miRNA	Radiation-Modulating Targets	Natural-Product-Regulated miRNA	Radiation-Associated miRNA	Radiation-Modulating Targets
miR-17-5p	[52]	DDIAS, MAP3K20, CCND2	miR-221-3p	[91]	RNF4, NIPBL, MDM2
miR-518b	[53]	EGR1	miR-23a-3p	[92]	MAP3K20, EGR1, RNF4
miR-223-3p	[52]	MDM2	miR-194-5p	[93]	SFRP2, YAP1, RNF4
miR-34c-5p	[54]	TMEM109, GATA3	miR-181a-5p	[94]	ATM, NIPBL, NUCKS1
miR-375-3p	[55]	MAP3K20	miR-34a-5p	[7,95]	TMEM109, GATA3
miR-877-3p	[56]	GTF2H5, KDM1A, WRN	miR-107	[103]	TSPYL5
miR-147a	[57]	MDM2	miR-3960	[95]	
miR-19b-3p	[58]	CCND2, MAP3K20	miR-217-3p	[61]	
miR-124-5p	[59]	DDIAS, YAP1, TLK2	miR-22-3p	[96,97]	
miR-182-5p	[178,303]	MAP3K20, CCND2	miR-126-3p	[52]	
miR-27a-3p	[60]	SFRP1, TLK2, GATA3, YAP1	miR-137-5p	[68]	
miR-217-5p	[61]	NIPBL, ZMPSTE24	miR-33a-5p	[98,99]	
miR-199a-5p	[62]	NUCKS1	miR-574-3p	[100]	
miR-92a-3p	[63]	RNF4, MAP3K20, NIPBL	miR-370-3p	[101]	
miR-18a-5p	[64]	ATM, RNF4, CCND2	miR-149-5p	[102]	
let-7a-5p	[65,66]	CCND2, TSPYL5, BCL2L1	miR-451a	[52]	
miR-9-5p	[67]	TMEM109, MAP3K20	miR-186-3p		GTF2H5, TSPYL5
miR-137-3p	[68]	MAP3K20, RNF4, KDM1A, NIPBL, NUCKS1	miR-204-3p		MAP3K20
miR-16-5p	[69,70]	CCND2, YAP1, NUCKS1	miR-29c-3p		CCND2, MDM2
miR-98-5p	[71]	CCND2, TSPYL5, BCL2L1	miR-132-3p		MAP3K20, EGR1, TSPYL5, NUCKS1
miR-30a-5p	[72]	NUCKS1	let-7f-1-3p		GATA3, SFRP2, MAP3K20, MDM2
miR-122-5p	[73]	MAP3K20	miR-542-3p		SFRP1
miR-155-5p	[74]	MAP3K20, GTF2H5, GATA3, MDM2	miR-590-5p		YAP1
miR-1271-5p	[75]	NIPBL, MAP3K20, CCND2	miR-192-5p		GTF2H5, NIPBL
miR-200c-3p	[76]	YAP1	miR-215-3p		TLK2, MAP3K20
miR-340-3p	[77]	MDM2	miR-215-5p		GTF2H5, NIPBL
miR-29b-3p	[78]	CCND2, MDM2	miR-371b-5p		MDM2, KDM1A
miR-211-5p	[79]	CCND2, NIPBL, GTF2H5, EGR1	miR-589-5p		RNF4
miR-15b-5p	[80]	CCND2, YAP1, NUCKS1	let-7c-5p		CCND2, TSPYL5, BCL2L1
miR-27b-3p	[81]	SFRP1, TLK2, GATA3, YAP1	miR-10b-5p		GATA3
miR-383-3p	[82]	CRYAB, CCND2, GATA3, NUCKS1, MAP3K20, MDM2	miR-3178		
miR-383-5p	[82]	ATR	miR-6085		
miR-146a-5p	[83]	NUCKS1, YAP1, RNF4	miR-375-5p		
miR-125b-5p	[84]	TLK2	miR-181c-3p		
miR-29a-3p	[85]	CCND2, MDM2	miR-129-1-3p		
miR-499a-5p	[86]	GATA3	miR-29b-1		
miR-186-5p	[87]	YAP1, NIPBL, NUCKS1	miR-99b-3p		
miR-145-5p	[88]	MDM2	miR-549a-5p		
miR-15a-5p	[89]	CCND2, YAP1, NUCKS1	miR-1247-5p		
miR-21-5p	[90]	YAP1	miR-137-5p		

miRNAs were derived from Table 1. Radiation-modulated genes were mined from miRDB based on these miRNAs (retrieval date: 1 June 2023).

**Table 3:** The exosome biogenesis-modulating effects and targets of natural-product-regulated miRNAs.

Natural-Product-Regulated miRNA	Exosome Biogenesis-Associated miRNA	Exosome Biogenesis-Modulating Targets	Natural-Product-Regulated miRNA	Exosome Biogenesis-Associated miRNA	Exosome Biogenesis-Modulating Targets
miR-199a-5p	[104,105]	ATP13A2, RAB7A, ATP9A	miR-3960	[143]	
miR-21-5p	[106]	RAB11A, MYO5B	miR-6085	[144]	
let-7a-5p	[107]	STEAP3, MYO5B	miR-18a-5p	[145]	
miR-9-5p	[108]	STEAP3, PDCD6IP, STAM, SDC1, SMPD3, CD34	miR-22-3p	[146]	
miR-30a-5p	[109]	RAB11A	miR-126-3p	[147]	
miR-1271-5p	[110]	RAB27A, RAB7A, MYO5B	miR-137-3p	[148]	
miR-29b-3p	[111]	SMPD3	miR-122-5p	[149]	
miR-223-3p	[112]	MYO5B, STAM	miR-23a-3p	[150]	
miR-383-3p	[113]	SMPD3, TSG101, SDCBP	miR-155-5p	[151]	
miR-371b-5p	[114]	RAB11A, STAM, SDCBP	miR-33a-5p	[152]	
miR-15a-5p	[115]	MYO5B, VPS4A	miR-574-3p	[153]	
miR-182-5p	[116]	RAB7A, ATP9A, SDC1	miR-132-3p	[154]	
miR-27a-3p	[117]	SMPD3	miR-211-5p	[155]	
miR-217-3p	[106]	VPS4B	miR-375-3p	[156]	
miR-92a-3p	[118,119]	VPS4B	miR-181c-3p	[157]	
miR-221-3p	[120]	PDCD6IP	miR-146a-5p	[158]	
miR-34a-5p	[121]	VPS4A	miR-542-3p	[159]	
miR-16-5p	[122]	MYO5B, VPS4A	miR-877-3p	[160]	
miR-186-3p	[123]	VPS4B	miR-192-5p	[161]	
miR-98-5p	[124]	STEAP3, MYO5B	miR-451a	[162]	
miR-17-5p	[125]	TSG101, MYO5B	miR-215-5p	[163]	
miR-200c-3p	[126]	PRKN, STAM	miR-99b-3p	[164]	
miR-29c-3p	[127]	SMPD3	miR-549a-5p	[165]	
miR-15b-5p	[128]	MYO5B, VPS4A	miR-1247-5p	[166]	
miR-27b-3p	[129]	SMPD3	miR-217-5p		ATP9A, STEAP3, PDCD6IP
miR-19b-3p	[124]	SDC1, VPS4B, MYO5B	miR-204-3p		RAB11A
miR-107	[130]	VPS4A, SDCBP	miR-340-3p		RAB11A
miR-125b-5p	[131]	VPS4B	let-7f-1-3p		SDCBP, RAB7A
miR-590-5p	[132]	RAB11A, MYO5B	miR-147a		ATP9A
miR-370-3p	[133]	ATP9A, RAB11A, RAB7A	miR-29b-1		COPS5
miR-194-5p	[93]	SDC4	miR-124-5p		PDCD6IP
miR-29a-3p	[134]	SMPD3	miR-3178		
miR-149-5p	[135]	VPS4A, CD34	miR-137-5p		
miR-499a-5p	[136]	ATP9A	miR-518b		
miR-186-5p	[137]	VPS4B, ATP9A, RAB27A, STAM	miR-375-5p		
miR-34c-5p	[138]	VPS4A	miR-383-5p		
miR-181a-5p	[139]	PDCD6IP, PRKN	miR-129-1-3p		
miR-145-5p	[140]	STAM	miR-215-3p		
let-7c-5p	[141]	STEAP3, MYO5B	miR-589-5p		
miR-10b-5p	[142]	SDC1, SMPD3	miR-137-5p		

miRNAs were derived from Table 1. Exosome biogenesis-modulating targets were mined from miRDB using these miRNAs (retrieval date: 1 June 2023).

**Table 4:** Radiation-modulating target-centric chart of natural product *-regulated miRNAs.

Natural Product	miRNA	Radiation-Modulated Gene	Natural Product	miRNA	Radiation-Modulated Gene	Natural Product	miRNA	Radiation-Modulated Gene
Chrysin	miR-18a-5p ↓	ATM	Lycopene	let-7f-1-3p	MAP3K20	Curcumin	miR-137-3p	RNF4
Vitamin C	miR-181a-5p		Daidzein	miR-122-5p		Quercetin	miR-146a-5p	
Piperine	miR-383-5p	ATR	Silymarin	miR-122-5p		Troxerutin	miR-146a-5p	
Chrysin	let-7a-5p	BCL2L1	Emodin	miR-1271-5p		Chrysin	miR-18a-5p ↓	
Quercetin	let-7a-5p		Hesperidin	miR-132-3p		Silymarin	miR-194-5p	
Withaferin A	let-7a-5p		Curcumin	miR-137-3p		Chrysin	miR-221-3p ↓	
Withaferin A	let-7c-5p		Diosmin	miR-155-5p ↓		Delphinidin	miR-23a-3p	
Curcumin	miR-98-5p		Genistein	miR-155-5p ↓		Melatonin	miR-23a-3p ↓	
Chrysin	let-7a-5p	CCND2	Melatonin	miR-155-5p ↓		Vitamin C	miR-589-5p ↓	
Quercetin	let-7a-5p		Diosmin	miR-17-5p ↓		Chrysin	miR-92a-3p	
Withaferin A	let-7a-5p		Fucoidan	miR-17-5p ↓		Mangiferin	miR-92a-3p	
Withaferin A	let-7c-5p		Piperine	miR-17-5p ↓		Lycopene	let-7f-1-3p	SFRP1
Emodin	miR-1271-5p		Berberine	miR-182-5p		Silymarin	miR-194-5p	
Apigenin	miR-15a-5p		Gallic acid	miR-182-5p		Betulinic acid	miR-27a-3p	
Vitamin D	miR-15a-5p		Matrine	miR-19b-3p		Genistein	miR-27a-3p	
Mangiferin	miR-15b-5p		Delphinidin	miR-204-3p		Lycopene	miR-27a-3p	
Curcumin	miR-16-5p		Vitamin C	miR-215-3p		Ligustrazine	miR-27a-3p	
Hesperidin	miR-16-5p		Delphinidin	miR-23a-3p		Withaferin A	miR-27a-3p ↓	
Ligustrazine	miR-16-5p		Melatonin	miR-23a-3p ↓		Mangiferin	miR-27b-3p	
Diosmin	miR-17-5p ↓		Parthenolide	miR-375-3p ↓		Resveratrol	miR-542-3p ↓	
Fucoidan	miR-17-5p ↓		Piperine	miR-383-3p		Withaferin A	miR-124-5p ↓	TLK2
Piperine	miR-17-5p ↓		Chrysin	miR-92a-3p		Resveratrol	miR-125b-5p ↓	
Berberine	miR-182-5p		Mangiferin	miR-92a-3p		Vitamin C	miR-215-3p	
Chrysin	miR-18a-5p ↓		Chrysin	miR-9-5p		Betulinic acid	miR-27a-3p	
Matrine	miR-19b-3p		Lycopene	let-7f-1-3p	MDM2	Genistein	miR-27a-3p	
Ligustrazine	miR-211-5p		Vitamin D	miR-145-5p		Ligustrazine	miR-27a-3p	
Thymol	miR-29a-3p		Troxerutin	miR-147a		Lycopene	miR-27a-3p ↓	
Fucoidan	miR-29b-3p		Diosmin	miR-155-5p ↓		Withaferin A	miR-27a-3p ↓	
Fucoidan	miR-29c-3p		Genistein	miR-155-5p ↓		Mangiferin	miR-27b-3p	
Piperine	miR-383-3p		Melatonin	miR-155-5p ↓		Chrysin	let-7a-5p	TSPYL5
Curcumin	miR-98-5p		Chrysin	miR-221-3p ↓		Quercetin	let-7a-5p	
Diosmin	miR-17-5p ↓	DDIAS	Genistein	miR-223-3p ↓		Withaferin A	let-7a-5p	
Fucoidan	miR-17-5p ↓		Thymol	miR-29a-3p		Withaferin A	let-7c-5p	
Piperine	miR-17-5p ↓		Fucoidan	miR-29b-3p		Parthenolide	miR-107	
Withaferin A	miR-124-5p ↓		Fucoidan	miR-29c-3p		Hesperidin	miR-132-3p	
Hesperidin	miR-132-3p	EGR1	Ferulic acid	miR-340-3p ↓		Curcumin	miR-186-3p	
Ligustrazine	miR-211-5p		Vitamin C	miR-371b-5p		Curcumin	miR-98-5p	
Delphinidin	miR-23a-3p		Piperine	miR-383-3p		Withaferin A	miR-124-5p ↓	YAP1
Melatonin	miR-23a-3p ↓		Emodin	miR-1271-5p	NIPBL	Quercetin	miR-146a-5p	
Gallic acid	miR-518b		Curcumin	miR-137-3p		Troxerutin	miR-146a-5p	
Lycopene	let-7f-1-3p	GATA3	Vitamin C	miR-181a-5p		Apigenin	miR-15a-5p	
Withaferin A	miR-10b-5p ↓		Ursolic Acid	miR-186-5p		Vitamin D	miR-15a-5p	
Diosmin	miR-155-5p ↓		Silymarin	miR-192-5p		Mangiferin	miR-15b-5p	
Genistein	miR-155-5p ↓		Ligustrazine	miR-211-5p		Curcumin	miR-16-5p	
Melatonin	miR-155-5p		Vitamin C	miR-215-5p		Hesperidin	miR-16-5p	
Betulinic acid	miR-27a-3p		Carvacrol	miR-217-5p		Ligustrazine	miR-16-5p	
Genistein	miR-27a-3p		Chrysin	miR-221-3p ↓		Ursolic Acid	miR-186-5p	
Lycopene	miR-27a-3p		Chrysin	miR-92a-3p		Silymarin	miR-194-5p	
Ligustrazine	miR-27a-3p		Mangiferin	miR-92a-3p		Ferulic acid	miR-200c-3p	
Withaferin A	miR-27a-3p ↓		Hesperidin	miR-132-3p	NUCKS1	Zingerone	miR-200c-3p	
Mangiferin	miR-27b-3p		Curcumin	miR-137-3p		3,3′-Diindolylmethane	miR-21-5p	
Chrysin	miR-34a-5p		Quercetin	miR-146a-5p		Diosmin	miR-21-5p	
Gallic acid	miR-34a-5p		Troxerutin	miR-146a-5p		Gallic acid	miR-21-5p ↓	
Hesperidin	miR-34a-5p		Apigenin	miR-15a-5p		Ginsenoside Rg1	miR-21-5p ↓	
Resveratrol	miR-34a-5p ↓		Vitamin D	miR-15a-5p		Hesperidin	miR-21-5p ↓	
Ursolic acid	miR-34c-5p		Mangiferin	miR-15b-5p		Matrine	miR-21-5p ↓	
Piperine	miR-383-3p		Curcumin	miR-16-5p		Quercetin	miR-21-5p ↓	
Ursolic acid	miR-499a-5p ↓		Hesperidin	miR-16-5p		Chrysin	miR-21-5p ↓	
Diosmin	miR-155-5p ↓	GTF2H5	Ligustrazine	miR-16-5p		Betulinic acid	miR-27a-3p	
Genistein	miR-155-5p ↓		Vitamin C	miR-181a-5p		Ligustrazine	miR-27a-3p	
Melatonin	miR-155-5p ↓		Ursolic acid	miR-186-5p		Genistein	miR-27a-3p ↓	
Curcumin	miR-186-3p ↓		Chlorogenic acid	miR-199a-5p		Lycopene	miR-27a-3p ↓	
Silymarin	miR-192-5p		Curcumin	miR-30a-5p		Withaferin A	miR-27a-3p ↓	
Ligustrazine	miR-211-5p		EGCG	miR-30a-5p		Mangiferin	miR-27b-3p	
Vitamin C	miR-215-5p		Piperine	miR-383-3p		Rutin	miR-590-5p	
Rutin	miR-877-3p		Chrysin	miR-34a-5p	TMEM109	Carvacrol	miR-217-5p	ZMPSTE24
Curcumin	miR-137-3p	KDM1A	Hesperidin	miR-34a-5p		Rutin	miR-877-3p	WRN
Rutin	miR-877-3p		Resveratrol	miR-34a-5p ↓		Piperine	miR-383-3p	CRYAB
Vitamin C	miR-371b-5p		Ursolic acid	miR-34c-5p				
			Chrysin	miR-9-5p				

* Natural products with modulating effects were derived from Table 1. ↓ indicates that miRNAs were downregulated by natural products, while miRNAs without ↓ were upregulated by natural products. Radiation-modulated genes were mined from miRDB based on these miRNAs (retrieval date: 1 June 2023).

**Table 5:** Exosome biogenesis-modulated target-centric chart of natural product *-regulated miRNAs.

Natural Product	miRNA	Exosome Biogenesis-Modulated Gene	Natural Product	miRNA	Exosome Biogenesis-Modulated Gene	Natural Product	miRNA	Exosome Biogenesis-Modulated Gene
Troxerutin	miR-147a	ATP9A	Delphinidin	miR-204-3p	RAB11A	Vitamin D	miR-145-5p	STAM
Berberine	miR-182-5p		3,3′-Diindolylmethane	miR-21-5p		Ursolic acid	miR-186-5p	
Gallic acid	miR-182-5p		Diosmin	miR-21-5p		Ferulic acid	miR-200c-3p	
Ursolic acid	miR-186-5p		Gallic acid	miR-21-5p ↓		Zingerone	miR-200c-3p	
Chlorogenic acid	miR-199a-5p		Ginsenoside Rg1	miR-21-5p ↓		Genistein	miR-223-3p ↓	
Carvacrol	miR-217-5p		Hesperidin	miR-21-5p ↓		Vitamin C	miR-371b-5p	
Sesamol	miR-370-3p		Matrine	miR-21-5p ↓		Chrysin	miR-9-5p	
Ursolic acid	miR-499a-5p ↓		Quercetin	miR-21-5p ↓		Chrysin	let-7a-5p	STEAP3
Chrysin	let-7a-5p	MYO5B	Chrysin	miR-21-5p ↓		Quercetin	let-7a-5p	
Quercetin	let-7a-5p		Curcumin	miR-30a-5p		Withaferin A	let-7a-5p	
Withaferin A	let-7a-5p		EGCG	miR-30a-5p		Withaferin A	let-7c-5p	
Withaferin A	let-7c-5p		Ferulic acid	miR-340-3p ↓		Carvacrol	miR-217-5p	
Emodin	miR-1271-5p		Sesamol	miR-370-3p		Chrysin	miR-9-5p	
Apigenin	miR-15a-5p		Vitamin C	miR-371b-5p		Curcumin	miR-98-5p	
Vitamin D	miR-15a-5p		Rutin	miR-590-5p		Diosmin	miR-17-5p ↓	TSG101
Mangiferin	miR-15b-5p		Emodin	miR-1271-5p	RAB27A	Fucoidan	miR-17-5p ↓	
Curcumin	miR-16-5p		Ursolic acid	miR-186-5p		Piperine	miR-17-5p ↓	
Hesperidin	miR-16-5p		Lycopene	let-7f-1-3p	RAB7A	Piperine	miR-383-3p	
Ligustrazine	miR-16-5p		Emodin	miR-1271-5p		Parthenolide	miR-107	VPS4A
Diosmin	miR-17-5p ↓		Berberine	miR-182-5p		Ursolic acid	miR-149-5p	
Fucoidan	miR-17-5p ↓		Gallic acid	miR-182-5p		Apigenin	miR-15a-5p	
Piperine	miR-17-5p ↓		Chlorogenic acid	miR-199a-5p		Vitamin D	miR-15a-5p	
Matrine	miR-19b-3p		Sesamol	miR-370-3p		Mangiferin	miR-15b-5p	
Diosmin	miR-21-5p		Withaferin A	miR-10b-5p ↓	SDC1	Curcumin	miR-16-5p	
Gallic acid	miR-21-5p ↓		Berberine	miR-182-5p		Hesperidin	miR-16-5p	
Ginsenoside Rg1	miR-21-5p ↓		Gallic acid	miR-182-5p		Ligustrazine	miR-16-5p	
Hesperidin	miR-21-5p ↓		Matrine	miR-19b-3p		Chrysin	miR-34a-5p	
Matrine	miR-21-5p ↓		Chrysin	miR-9-5p		Hesperidin	miR-34a-5p	
Quercetin	miR-21-5p ↓		Silymarin	miR-194-5p	SDC4	Resveratrol	miR-34a-5p ↓	
Chrysin	miR-21-5p ↓		Withaferin A	miR-10b-5p ↓	SMPD3	Ursolic acid	miR-34c-5p	
Genistein	miR-223-3p ↓		Betulinic acid	miR-27a-3p		Resveratrol	miR-125b-5p ↓	VPS4B
Rutin	miR-590-5p		Mangiferin	miR-27a-3p		Curcumin	miR-186-3p	
Curcumin	miR-98-5p		Ligustrazine	miR-27a-3p		Ursolic acid	miR-186-5p	
Carvacrol	miR-217-5p	PDCD6IP	Genistein	miR-27a-3p ↓		Matrine	miR-19b-3p	
Chrysin	miR-221-3p ↓		Withaferin A	miR-27a-3p ↓		Carvacrol	miR-217-3p	
Chrysin	miR-9-5p		Mangiferin	miR-27b-3p		Chrysin	miR-92a-3p	
Vitamin C	miR-181a-5p		Silymarin	miR-29a-3p		Mangiferin	miR-92a-3p	
Withaferin A	miR-124-5p ↓		Fucoidan	miR-29b-3p		Chrysin	miR-9-5p	CD34
Lycopene	let-7f-1-3p	SDCBP	Fucoidan	miR-29c-3p		Ursolic acid	miR-149-5p	
Parthenolide	miR-107		Piperine	miR-383-3p		Vitamin C	miR-181a-5p	PRKN
Piperine	miR-383-3p		Chrysin	miR-9-5p		Ferulic acid	miR-200c-3p	
Vitamin C	miR-371b-5p		Vitamin C	miR-29b-1 ↓	COPS5	Zingerone	miR-200c-3p	
						Chlorogenic acid	miR-199a-5p	ATP13A2

* Natural products with modulating effects were derived from Table 1.↓ indicates that miRNAs were downregulated by natural products, while miRNAs without ↓ were upregulated by natural products (retrieval date: 1 June 2023).

## Data Availability

No new data were created.
